# Prognostic Factors and Effect Modifiers in Patients With Relapse or Refractory Diffuse Large B‐Cell Lymphoma After Two Lines of Therapy: A Systematic Literature and Expert Clinical Review

**DOI:** 10.1111/ejh.14423

**Published:** 2025-05-09

**Authors:** Bastian von Tresckow, Pau Abrisqueta, Irene Zamanillo, Ángel Serna, Yuting Kuang, Jennifer Uyei, Mohsin Shah, Laura Walsh, Eileen Thorley, Krystal Cantos, Emaan Rashidi, Christian Hampp, Jessica J. Jalbert, Alexi N. Archambault, Yingxin Xu, Shivani Aggarwal, Srikanth Ambati, Hesham Mohamed, Qiufei Ma, Ana Jiménez‐Ubieto

**Affiliations:** ^1^ Department of Hematology and Stem Cell Transplantation, West German Cancer Center and German Cancer Consortium (DKTK Partner Site Essen), University Hospital Essen University of Duisburg‐Essen Essen Germany; ^2^ Department of Hematology Vall d'Hebron University Hospital, Vall d'Hebron Institute of Oncology Barcelona Spain; ^3^ Hematology Department University Hospital 12 de Octubre Madrid Spain; ^4^ IQVIA Inc. Durham North Carolina USA; ^5^ Regeneron Pharmaceuticals Inc. Tarrytown New York USA

**Keywords:** comparative effectiveness research, diffuse large B‐cell lymphoma, systematic review

## Abstract

**Objectives:**

The objective of this systematic literature review (SLR) combined with expert clinical review was to identify and rank prognostic factors and effect measure modifiers (EMMs) systematically and comprehensively in patients with relapsed or refractory (R/R) diffuse large B‐cell lymphoma (DLBCL) who initiate treatment after ≥ 2 prior lines of therapy (LoTs; 3L+ R/R DLBCL).

**Methods:**

We performed an SLR of studies published between 2016 and 2021 and extracted study characteristics, prognostic factors, and EMMs. This was followed by clinical review and ranking of findings by subject matter experts using questionnaires, follow‐up interviews, and quantitative ranking.

**Results:**

Across 46 included studies, the SLR identified 36 prognostic factors significantly associated with ≥ 1 clinical outcome. Based on subject matter expert ranking of the SLR‐derived list, the five most important prognostic variables in descending order are: early chemo‐immunotherapy failure, Eastern Cooperative Oncology Group performance status, refractory to last LoT, number of prior LoTs, and double‐ or triple‐hit lymphoma.

**Conclusions:**

This SLR and expert clinical review is the first to provide a comprehensive assessment of prognostic factors for 3L+ R/R DLBCL. No statistically significant EMMs were identified. This robust multi‐method approach can assist in selecting prognostic variables for comparative analyses between real‐world studies and clinical trials.

## 1 Introduction

Diffuse large B‐cell lymphoma (DLBCL) is the most common subtype of non‐Hodgkin lymphoma [1, 2]. Despite the effectiveness of chemo‐immunotherapy in the first‐line treatment setting, 40% of patients with DLBCL are either refractory to the treatment or will relapse after achieving complete response (CR) [3, 4, 5]. Considering the relatively limited treatment options and generally poor outcomes, there is an unmet medical need to identify effective treatments for patients with relapsed or refractory (R/R) DLBCL, particularly for those requiring third‐line treatment and above (3L+).

Since 2018, chimeric antigen receptor (CAR) T‐cell therapy has significantly altered the treatment landscape for DLBCL, initially for patients with multiple relapses and more recently for those in earlier lines of therapy (LoTs) with proven efficacy and an acceptable safety profile. However, a substantial number of patients still experience relapse following therapy [6]. The use of CAR T‐cell therapy remains impeded by several factors, including patient eligibility, rapidly progressing diseases, lengthy manufacturing time, need for bridging therapies, treatment‐related toxicities (i.e., cytokine release syndrome and immune effector cell‐associated neurotoxicity syndrome), and financial challenges due to the high cost that may not be affordable in certain healthcare settings [5]. Other novel treatments, such as bispecific antibodies like epcoritamab and glofitamab, have also emerged as additional treatment options for patients with R/R DLBCL [1].

The use of external controls has been employed to contextualize single‐arm trials of newer therapies in DLBCL (axicabtagene ciloleucel: SCHOLAR‐1, tafasitamab: RE‐MIND, and lisocabtagene maraleucel: NDS‐NHL‐001) [7, 8, 9]. These external controls can serve as a point of reference for comparing the results with clinical trials. Valid comparisons require balanced prognostic factors between trial participants and external controls, and an understanding of effect measure modifiers (EMMs) to inform the design of subgroup analyses. Such variables should be identified through a systematic review and pre‐specified in a study protocol [10]. Given that sample‐size considerations may preclude adjustment for all prognostic variables, ranking them by their importance can help researchers focus on the most critical prognostic variables. This approach is aligned with guidance provided by the Institute for Quality and Efficiency in Health Care [10], which requires that relevant confounders be pre‐specified in the study protocol [10].

The objective of this systematic literature review (SLR) combined with expert clinical review was to identify and rank prognostic factors and EMMs systematically and comprehensively in patients with R/R DLBCL after two LoTs.

## 2 Methods

This study was conducted in two stages. The first stage consisted of SLR‐based identification of prognostic factors, followed by a second stage encompassing a clinical review and ranking of SLR findings by subject matter experts. The SLR followed the industry‐accepted guidelines described by the Cochrane Handbook for Systematic Reviews of Interventions [11] and the Preferred Reporting Items for Systematic Reviews and Meta‐Analyses (PRISMA) [12]. Guidelines from the European Medicines Agency [13], U.S. Food and Drug Administration [14], Institute for Quality and Efficiency in Health Care (IQWiG) [10], and UK National Institute for Health and Care Excellence [15, 16] were also reviewed for SLR methodology, as applicable.

A detailed protocol was developed prior to conducting the review, and the review was registered a priori in PROSPERO (registration ID: CRD42022307557).

### 2.1 Search Strategy

Comprehensive literature searches were conducted using MEDical Literature Analysis and Retrieval System Online, Excerpta Medica Database, and Cochrane Central Register of Controlled Trials between January 1, 2016, and December 13, 2021 (complete search strategies are presented in Supporting Information S1: Appendix A). Searches were supplemented by conference abstract reviews for the American Society of Clinical Oncology, the European Society for Medical Oncology (ESMO), the American Society of Hematology, and the European Hematology Association conferences in 2021. Forward citation searches were undertaken using Google Scholar, based on 10 included references. The bibliographies of four recently published reviews on the related topic area, as well as ESMO and National Comprehensive Cancer Network guidelines, were also reviewed to identify additional relevant studies [17, 18, 19, 20, 21, 22].

### 2.2 Eligibility Criteria

The scope of the research and patient, intervention, comparison, outcome, time, and setting (PICOTS) criteria [11] for including and excluding studies are outlined in Supporting Information S1: Appendix B.

### 2.3 Study Selection, Data Collection, and Risk of Bias Assessment

Unique records identified by the search were screened for eligibility by title and abstract, which was followed by full‐text screening by two reviewers working independently. When there was uncertainty about inclusion/exclusion criteria, or there was a discrepancy between the two reviewers, a third reviewer adjudicated a decision to include or exclude. Data were collected from the eligible studies and entered into an Excel workbook for synthesis. For each study, key study methodological characteristics, patient characteristics, and results were extracted and tabulated. Data extraction of numeric values was conducted independently by two investigators, and the source document was checked by a third reviewer for any discrepancies. Risk of bias assessment of individual studies was performed using the quality in prognostic studies (QUIPS) tool (Supporting Information S1: Appendix C) [23].

### 2.4 Data Synthesis

All eligible studies were included to describe the prognostic factors and/or EMMs reported for individual clinical outcomes. Results were synthesized narratively by the type of prognostic factors and EMMs, with findings tabulated.

### 2.5 Clinical Review

Following the conduct of the SLR, the identified potential prognostic variables were evaluated by the study team to remove treatment‐specific factors (e.g., those related to stem cell transplantation [SCT]) and post‐baseline variables, determine their availability in the single‐arm trial and in real‐world data (RWD), and develop a questionnaire.

In the questionnaire (Supporting Information S1: Appendix D), prognostic variables were grouped by type of variable—patient demographics and clinical characteristics, disease characteristics, prior treatment characteristics, and imaging and laboratory values. Each prognostic variable was reviewed by an international panel comprising three clinical experts in the field of lymphoma who categorized the prognostic impact on treatment response and survival on a 5‐point scale ranging from (*very high importance*) to (*not important*). A comprehensive approach was taken for the ranking of variables, that is, clinical experts were asked to categorize variables in terms of their prognostic impact on treatment response and survival. The clinicians were asked to consider possible correlations among the variables, consider possible effect modifiers, provide variable definitions (e.g., early chemo‐immunotherapy failure), and assess whether there were any other prognostically important variables not captured in the questionnaire. For each variable, the clinical experts categorized the availability within RWD on a 3‐point scale ranging from (*readily available*) to (*limited availability*).

The results of the three expert‐completed questionnaires were reviewed, and the top 10 most important variables were determined by summing the clinicians' categorization of prognostic impact across the questionnaires and considering variable availability in the event of a tie. Individual interviews with each clinical expert were held to clarify variables and definitions, discuss discrepant categorization and the rationale for their decisions, and rank the prognostic variables from 1 to 10. Following the three interviews, the ranking of each variable was summed across the three experts to determine the final ranking. In the event of a tie, the variables were assigned the same rank.

## 3 Results

### 3.1 Studies Identified

**FIGURE 1:**
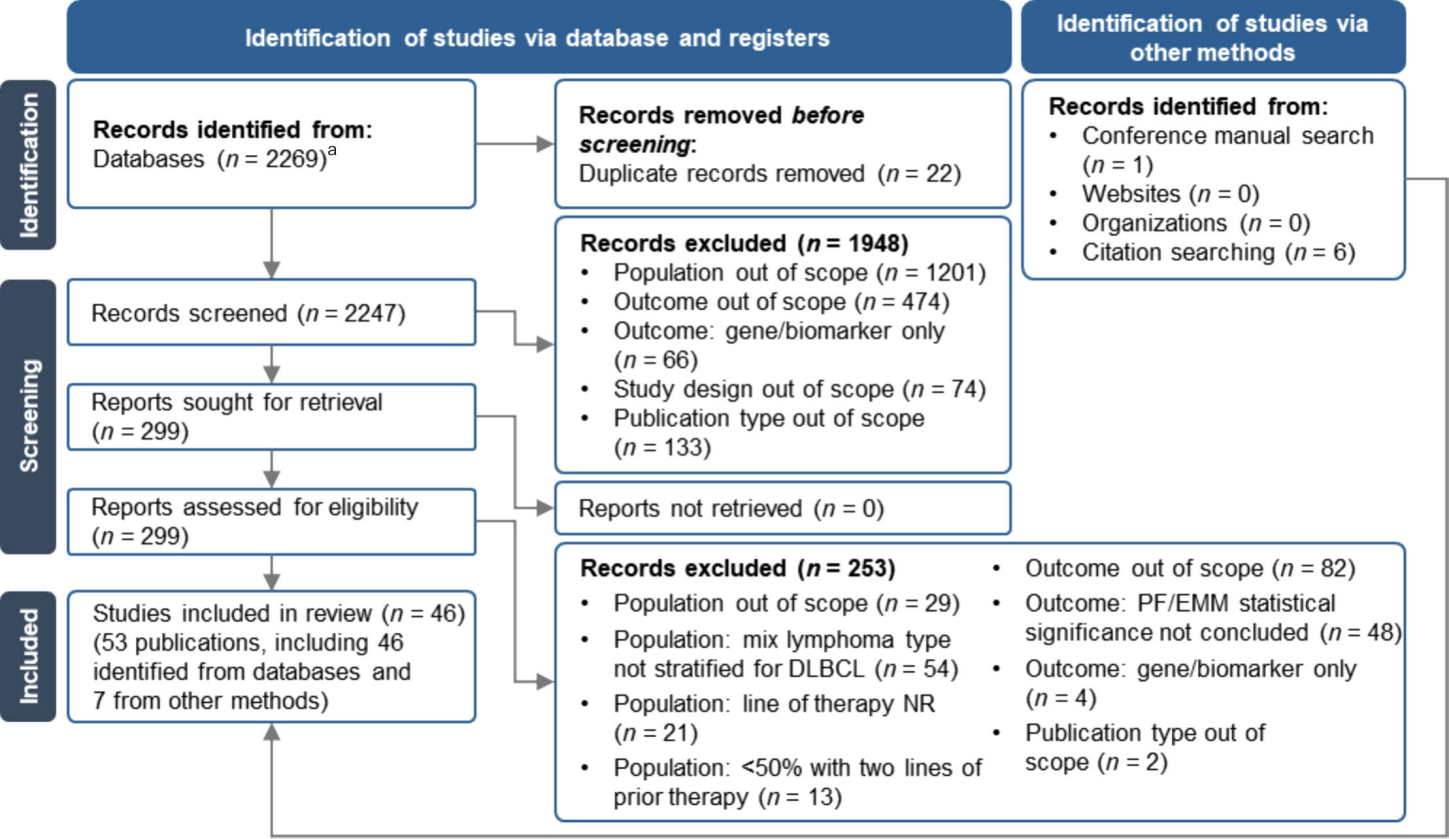
PRISMA flow diagram. DLBCL = diffuse large B‐cell lymphoma; EMM = effect measure modifier; NR = not reported; PF = prognostic factor. ^a^Search was conducted on December 13, 2021.

The database searches identified a total of 2269 records. Following deduplication, 2247 records underwent title and abstract screening, of which 299 records were retained for full‐text review. Following full‐text review, 46 records meeting the eligibility criteria were included. Seven additional records were identified by other methods. Overall, 53 publications [24, 25, 26, 27, 28, 29, 30, 31, 32, 33, 34, 35, 36, 37, 38, 39, 40, 41, 42, 43, 44, 45, 46, 47, 48, 49, 50, 51, 52, 53, 54, 55, 56, 57, 58, 59, 60, 61, 62, 63, 64, 65, 66, 67, 68, 69, 70, 71, 72, 73, 74, 75, 76] (24 journal articles and 29 conference abstracts) reporting data on 46 studies were included in the review. The process of searching and screening was summarized in a PRISMA flow diagram (Figure 1).

### 3.2 Study and Patient Characteristics

Characteristics of the included studies are summarized in Table S1 (Supporting Information S1: Appendix E). Among the 46 included studies, 32 unique population sources were involved. Five studies [24, 27, 28, 29, 30] were conducted using data from two randomized controlled trials, four studies [31, 32, 34, 35] were conducted using data from three non‐randomized controlled trials, and 37 observational studies [36, 37, 38, 39, 40, 41, 42, 43, 44, 45, 46, 47, 48, 49, 50, 51, 52, 53, 54, 55, 56, 57, 58, 59, 60, 61, 62, 64, 65, 66, 67, 68, 69, 70, 71, 75, 76] were based on data from clinical centers or registries. In this review, clinical trials and observational studies were considered equal grade in the evidence synthesis. Some studies included patients from the same trial or overlapping data sources, but all studies were all included to capture important subpopulations and to ensure comprehensiveness. The sample size across the included studies varied from 17 [35] to 6947 [75], and the median follow‐up time ranged from 4 [60] to 127 [62] months. The intervention type was SCT in 12 studies [42, 45, 50, 52, 61, 62, 64, 69, 70, 71, 75, 76], CAR T‐cell therapy in eight studies [34, 36, 38, 41, 46, 55, 58, 59], polatuzumab vedotin‐based therapy in six observational studies [37, 43, 44, 47, 49, 60], selinexor in four studies [24, 27, 29, 30], ibrutinib‐based therapy in three studies [31, 32, 35], and combination chemotherapy in eight studies [28, 53, 54, 65, 66, 67, 68]. The type of treatment was not reported in five studies [39, 40, 51, 56, 57]. Among the 46 studies, all patients had at least two prior LoTs in 21 studies, eight studies included populations in which at least 50% of the study population received at least two prior LoTs, and 17 studies included populations that had a median/mean of at least two prior LoTs. A total of 45 studies included only patients with DLBCL, whereas one study included two lymphoma types but had results stratified for the DLBCL population. Across the included study treatment arms, the median age ranged from 40 [74] to 72 [43] years, and the proportion of males varied from 33% [65] to 75% [28] across the studies. Among the 46 studies, only 10 studies [34, 37, 40, 45, 49, 52, 54, 62, 69, 73, 76] reported race/ethnicity information; four studies [34, 37, 49, 73, 76] were conducted in an Asian population, and six studies [40, 45, 52, 54, 62, 69] were conducted in a mixed population that was predominantly White (> 80%).

### 3.3 Quality Assessment of Included Studies

Risk of bias was assessed using the QUIPS tool [23]. Most of the included studies had a high or moderate risk of bias due to lack of reporting, specifically in the “Study Attrition” and “Statistical Analysis and Reporting” domains. A graphical summary of the risk of bias assessment can be found in Supporting Information S1: Appendix F.

### 3.4 Prognostic Factors and EMMs

#### 3.4.1 SLR

All 46 studies reported statistically significant associations (*p* < 0.05) between variables and clinical outcomes of interest. None of the studies identified statistically significant EMMs. Thirty‐six prognostic factors were identified to have statistically significant associations with seven clinical outcomes, including overall survival (OS; 38 studies), progression‐free survival (PFS; 22 studies), objective response rate (eight studies), non‐relapse mortality (four studies), CR (three studies), relapse/progression (two studies), and duration of response (one study). The prognostic variables were categorized into four groups: patient demographics and clinical characteristics, disease characteristics, treatment characteristics, and imaging and laboratory values. The associated clinical outcomes, directionality of the association, and study counts for each prognostic factor are summarized in Tables 1, 2, 3, 4. The prognostic factors and their characteristics were captured as reported by the studies, with additional information for effect estimates and supporting evidence presented in Table S2 (Supporting Information S1: Appendix E). Among the identified prognostic factors, higher Eastern Cooperative Oncology Group (ECOG) performance status, older age, having (primary) refractory disease, not achieving response to current or prior therapy, higher International Prognostic Index (IPI) composite score, and elevated lactate dehydrogenase (LDH) levels were associated with worse outcomes in five or more studies (OS, 29; PFS, 13; others, eight). Four variables (age, transformed disease, non‐germinal center B‐cell DLBCL, and LDH level) had evidence of conflicting associational directions.

**TABLE 1:** Patient demographics and clinical characteristics—Summary of study count, directionality, example characteristics, and affected outcomes for statistically significant prognostic factors.

Prognostic factor	Study count	Directionality—characteristics associated with worse outcomes	Example characteristics (vs. reference)—category with favorable outcomes in bold	Clinical outcomes with study counts
ECOG performance status	*N* = 9^a^	Higher ECOG performance status	≥ 2 (vs. **< 2**) 3–4 (vs. **0–2**)	OS: 9,^a^ PFS: 1
Age^b^	*N* = 7^a^	Older age in five studies; younger age in two studies	≥ 65 years (vs. **< 65 years**) ≥ 55 years (vs. **15–39 years**) **> 50 years** (vs. younger)	OS: 5,^a^ PFS: 1, NRM: 1, overall mortality: 1, CR: 1
KPS	*N* = 3	Lower KPS	< 80% (vs. **80%–100%**) **Admittance KPS status as continuous variable**	OS: 3, PFS: 1, relapse/progression: 1
Presence of comorbidities^c^	*N* = 1	Presence of significant comorbidities	Significant comorbidities (vs. **without significant comorbidities**)	OS: 1, PFS: 1

Abbreviations: CIRS = Cumulative Illness Rating Scale; CR = complete response; ECOG = Eastern Cooperative Oncology Group; KPS = Karnofsky performance status; NRM = non‐relapse mortality; OS = overall survival; PFS = progression‐free survival.

^a^ Two studies used the same data source and may have overlapping populations.

^b^ Prognostic factor with conflicting directionality across studies.

^c^ Presence of comorbidities: assessed by CIRS; significant comorbidities were defined as CIRS total score of ≥ 7 or CIRS score of 3 or 4 in ≥ 1 organ system [59].

**TABLE 2:** Disease characteristics—Summary of study count, directionality, example characteristics, and affected outcomes for statistically significant prognostic factors.

Prognostic factor	Study count	Directionality—characteristics associated with worse outcomes	Example characteristics (vs. reference)—category with favorable outcomes in bold	Clinical outcomes with study counts
Refractory disease	*N* = 10^a^	Refractory disease	Refractory disease (vs. **non‐refractory disease**) Primary refractory disease^b^ (vs. **relapsed disease**)	OS: 5, PFS: 7,^a^ CR: 2, ORR: 2
R‐IPI or t‐IPI	*N* = 5	Higher IPI score	3–5 (vs. **0–2**) 4–5 (vs. **0–1**)	OS: 5, ORR: 1
Double‐expressor or double‐hit lymphoma^c^	*N* = 4	Double‐expressor or double‐hit lymphoma	Double/triple expressor (vs. **no**) DEL (vs. **non‐DEL**) DEL (vs. **neither DEL nor DHL**) DHL (vs. **neither DEL nor DHL**) Both DEL and DHL (vs. **non‐DEL/non‐DHL**; **DEL**)	OS: 3, PFS: 1, NRM: 1, ORR: 2^a^
MYC overexpression or mutation^d^	*N* = 3	Overexpression or mutation of MYC	MYC mutation (vs. **others**) MYC rearrangement (positive on FISH vs. **negative**) cMYC ≥ 40% tumor‐positive cells (vs. **cMYC < 40% tumor‐positive cells**)	OS: 2, ORR: 1
Transformed disease^e^	*N* = 3	Transformed disease in two studies; de novo disease in one study	Transformed from follicular lymphoma (vs. **de novo**) **Transformed (history unspecified)** (vs. de novo)	OS: 1, PFS: 1, DOR: 1
Cell of origin^e^	*N* = 2	Non‐GCB and GCB in one study each	Non‐GCB (vs. **GCB**) **Non‐GCB** (vs. GCB)	OS: 1, ORR: 1
Ann Arbor stage	*N* = 2	Higher Ann Arbor stage	III–IV (vs. **I–II**)	OS: 2
Disease bulk	*N* = 2	Bulky disease	> 7.5 cm (vs. **≤ 7.5 cm**) Yes (vs. **no**)	PFS: 1, ORR: 1
Time from diagnosis	*N* = 2	Shorter time from diagnosis	Shorter (vs. **longer**) **Days from diagnosis to ASCT (as continuous variable)**	ORR: 1, CR (after relapse/progression): 1
Tumor burden^f^	*N* = 1	High tumor burden	High (vs. **low**)	PFS: 1, ORR: 1
BCL2 expression	*N* = 1	Overexpression of BCL2	Positive (vs. **negative**)	OS: 1, PFS: 1
Year of diagnosis of DLBCL	*N* = 1	Diagnosis of DLBCL before 2002	Before 2002 (vs. **after 2002**)	OS: 1, PFS: 1, NRM: 1
B symptoms	*N* = 1	Presence of B symptoms	Yes (vs. **no**)	OS: 1

Abbreviations: ASCT = autologous stem cell transplantation; BCL2 = B‐cell lymphoma 2; CR = complete response; DEL = double‐expressor lymphoma; DHL = double‐hit lymphoma; DLBCL = diffuse large B‐cell lymphoma; DOR = duration of response; FISH = fluorescence in situ hybridization; GCB = germinal center B‐cell like; IPI = International Prognostic Index; NRM = non‐relapse mortality; ORR = objective response rate; OS = overall survival; PFS = progression‐free survival; R‐IPI = revised International Prognostic Index; t‐IPI = tertiary International Prognostic Index.

^a^ Two studies used the same data source and may have overlapping populations.

^b^ Primary refractory disease: defined as a duration of first remission of < 12 months in one study [68], but no additional definition was provided in other studies.

^c^ DEL or DHL: DEL was defined as the dual expression of MYC and BCL2 proteins; DHL was defined as concurrent rearrangements of MYC and BCL2 and/or BCL6.

^d^ MYC overexpression or mutation: MYC overexpression was defined as ≥ 40% positive cells in one study [74]. MYC positivity was determined by FISH in one study [59], which indicates MYC rearrangement in the context of DLBCL. MYC mutation was reported in one study [56] without further specification of whether it pertained to rearrangement specifically.

^e^ Prognostic factor with conflicting directionality across studies.

^f^ High tumor burden: defined as the maximum dimension of largest tumor of ≥ 4 cm or metabolic tumor volume of ≥ 100 cm^3^ [31].

**TABLE 3:** Treatment characteristics—Summary of study count, directionality, example characteristics, and affected outcomes for statistically significant prognostic factors.

Prognostic factor	Study count	Directionality—characteristics associated with worse outcomes	Example characteristics (vs. reference)—category with favorable outcomes in bold	Clinical outcomes with study counts
Response to current therapy	*N* = 8^a^	Not achieving CR/PR to current therapy	No response (SD/PD) (vs. **CR/PR**) PD (vs. **CR/PR**)	OS: 7,^a^ PFS: 1
Response to prior therapy	*N* = 5^b^	Not achieving CR to therapy prior to SCT	*Response to therapy prior to SCT (remission status at transplantation)* Non‐CR (vs. **CR**) PR (vs. **CR**)	OS: 5,^b^ PFS: 3, NRM: 1, CR: 1, relapse/progression: 2
	*N* = 1	Not achieving response to prior 3L treatment	*Response to prior 3L treatment* SD/PD (vs. **CR/CRu**) SD/PD (vs. **PR**)	OS: 1
Prior LoTs	*N* = 4	Greater number of prior LoTs	Per extra LoT/treatment (as continuous variable) ≥ 2 (vs. **< 2**)	OS: 1, PFS: 3
Conditioning regimen	*N* = 2^b^	TBI or myeloablative conditioning	TBI conditioning (vs. **no TBI conditioning**) Myeloablative (vs. **reduced intensity conditioning/non‐myeloablative conditioning**)	OS: 1, PFS: 1, NRM: 2,^b^ overall mortality: 1
Prior ASCT	*N* = 2	Not receiving prior ASCT	No (vs. **yes**)	OS: 1, PFS: 1, ORR: 1
Time from ASCT to relapse/progression	*N* = 2	Shorter time from ASCT to relapse/progression	≤ 1 year (vs. **> 1 year**) **Days from ASCT to relapse/progression (as continuous variable)**	OS: 2, PFS: 1, CR: 1
Early chemo‐immunotherapy failure^c^	*N* = 1	Early chemo‐immunotherapy failure	Yes (vs. **no**)	OS: 1
Primary intent of radiation therapy	*N* = 1	Radiation delivered for palliative symptoms or bridging to another therapy	Palliative symptoms (vs. **salvage**) Bridge (vs. **salvage**)	OS: 1
CAR T‐cell therapy eligibility	*N* = 1	Not being eligible for CAR T‐cell therapy	Not eligible (vs. **eligible**)	OS: 1
Post‐ASCT disease‐free interval	*N* = 1	Shorter post‐ASCT disease‐free interval	< 6 months (vs. **≥ 12 months**)	OS: 1
Graft type	*N* = 1	Bone marrow grafts	Bone marrow (vs. **peripheral blood**)	OS: 1
Time from autologous to allogeneic HCT	*N* = 1	Shorter interval	< 12 months (vs. **≥ 12 months**)	PFS: 1, relapse/progression: 1
Type of donor	*N* = 1	Unrelated donor transplantation	URD/HLA‐identical sibling (vs. **well‐matched/partially matched**)	NRM: 1

Abbreviations: 3L = third line; ASCT = autologous stem cell transplantation; CAR = chimeric antigen receptor; CR = complete response; CRu = complete response unconfirmed; HCT = hematopoietic cell transplantation; HLA = human leukocyte antigen; LoT = line of therapy; NRM = non‐relapse mortality; ORR = objective response rate; OS = overall survival; PD = progressive disease; PFS = progression‐free survival; PR = partial response; SCT = stem cell transplantation; SD = stable disease; TBI = total body irradiation; URD = unrelated donor.

^a^ Several studies used the same data sources and may have overlapping populations.

^b^ Two studies used the same data source and may have overlapping populations.

^c^ Early chemo‐immunotherapy failure: defined as patients with primary refractory disease or relapse within 12 months of diagnosis [45].

**TABLE 4:** Imaging and lab measures—Summary of study count, directionality, example characteristics, and affected outcomes for statistically significant prognostic factors.

Prognostic factor	Study count	Directionality—characteristics associated with worse outcomes	Example characteristics (vs. reference)—category with favorable outcomes in bold	Clinical outcomes with study counts
LDH^a^	*N* = 7	Elevated LDH in six studies; lower LDH in one study	Elevated (vs. **normal**) > 450 U/L (vs. **≤ 450 U/L**) **LDH (as continuous variable)** ^a^	OS: 6, PFS: 1
Deauville score^b^	*N* = 4	Higher Deauville score	> 3 (vs. **≤ 3**) Grade 5 (**vs. Grade 1–4**)	OS: 3, PFS: 3
SUV_max_ on pre‐transplant PET/CT	*N* = 2	Higher SUV_max_	> 17.1 (vs. **< 17.1**) SUV_max_ (as continuous variable)	OS: 1, response (not defined): 1
ALC/AMC	*N* = 2	Lower ALC, lower ALC/AMC ratio, or high‐risk ALC/AMC prognostic score	ALC < 1000/μL (vs. **ALC ≥ 1000/μL**) ALC/AMC ratio ≤ 1.5 (vs. **> 1.5**) ALC/AMC prognostic score as high‐risk (vs. **low‐risk; intermediate‐risk**)	OS: 2, ORR: 1
TLG value on pre‐transplant PET/CT	*N* = 1	Higher TLG value	TLG value (as continuous variable) High (vs. **low**)	OS: 1, PFS: 1
Minimal residual disease detected by circulating tumor DNA	*N* = 1	Positive minimal residual disease	Positive (vs. **negative**)	OS: 1, PFS: 1

Abbreviations: ALC = absolute lymphocyte count; AMC = absolute monocyte count; CT = computed tomography; LDH = lactate dehydrogenase; ORR = objective response rate; OS = overall survival; PET = positron emission tomography; PFS = progression‐free survival; SUV_max_ = maximum standardized uptake value; TLG = total lesion glycolysis.

^a^ Prognostic factor with conflicting directionality across studies.

^b^ The Deauville 5‐point scale is based on a visual comparison between the uptake of lymphoma tissue and that of the liver and mediastinum in PET/CT.

#### 3.4.2 Clinical Review

During the questionnaire and following individual interviews, no prognostic factors were considered missing by the clinical experts, and discussions were held with the experts to address discrepancies in grading from the questionnaire. The final ranked list of the 10 most important prognostic variables in descending order of importance was as follows: early chemo‐immunotherapy failure, ECOG performance status, refractory to last LoT, number of prior LoTs, double‐ or triple‐hit lymphoma, age at start of LoT, IPI risk classification, Ann Arbor disease stage, serum LDH, and Deauville score (Table 5).

**TABLE 5:** Final ranked prognostic variables based on expert clinical review.

Variable	Rankings			Sum of rankings	Final ranking
	Clinical Expert 1	Clinical Expert 2	Clinical Expert 3		
Early chemo‐immunotherapy failure^a^	2	1	3	6	1
ECOG performance status	5	2	1	8	2
Refractory to last LoT^b^	1	5	2	8	2
Number of prior LoTs	3	6	7	16	4
Double‐ or triple‐hit lymphoma	4	7	6	17	5
Age at start of LoT	9	3	5	17	5
IPI risk classification	6	10	4	20	7
Ann Arbor disease stage	7	8	8	23	8
Serum LDH	10	4	9	23	8
Deauville score	8	9	10	27	10

*Note*: Variables were assessed before each LoT if not otherwise specified.

Abbreviations: ECOG = Eastern Cooperative Oncology Group; IPI = International Prognostic Index; LDH = lactate dehydrogenase; LoT = line of therapy.

^a^ Defined in the questionnaire as no complete response after the first LoT or relapse or progression within 12 months of initial diagnosis. Following clinical expert discussions, defined as primary refractory or relapse within 12 months of the first LoT with the following options: primary refractory (no response or early relapse [i.e., within < 6 months]), relapse within 6–12 months, or response and no relapse within the first 12 months.

^b^ Following clinical expert discussions, defined as no response (stable disease or progressive disease) or relapse within 6 months of completion of the most recent LoT.

## 4 Discussion

The use of single‐arm trials has allowed transformative therapies to be made more expeditiously available to patients with diseases that have high unmet needs. However, contextualizing the findings of these trials using external controls requires the identification of prognostic factors and pre‐specification of variables for confounder adjustment. Despite the availability of multiple studies on prognostic factors across various diseases, they are often of variable quality and have inconsistent findings. The German IQWiG guidelines [77] suggest using SLR combined with an expert review of its results as a recommended method for identifying and pre‐specifying prognostic variables and EMMs to support the use of RWD‐derived external control arms. To the best of our knowledge, this is the first study combining SLR‐based identification of prognostic factors with a clinical review by subject matter experts to systematically and comprehensively identify and rank prognostic factors and EMMs in patients with R/R DLBCL after two LoTs. A total of 36 disease and treatment characteristics were found to be important prognostic factors of clinical outcomes for patients with R/R DLBCL, as reported in the literature (see Table S2 in Supporting Information S1: Appendix E). However, no statistically significant EMMs were identified based on the SLR.

This review captured the most recent evidence published since 2016, and it is the first SLR focusing on the 3L+ R/R DLBCL patient population. In comparison with prior reviews and published indices on the prognostic factors for DLBCL [78, 79, 80, 81, 82], this review confirmed that several disease and treatment characteristics, as well as lab measures, are important prognostic factors of clinical outcomes in patients with 3L+ R/R DLBCL. This review identified several additional prognostic factors, such as Karnofsky performance status, refractory disease, response to prior therapy, number of prior LoTs, prior autologous stem cell transplantation (ASCT), use of myeloablative conditioning regimen, and time from ASCT to relapse/progression of disease.

To ensure clinical context and a holistic approach, an international panel of expert clinicians reviewed the list of the 10 most significant prognostic variables identified through SLR. The decision to have a single, consolidated list of prognostic variables for all outcomes was based on the fact that it is not possible to pre‐specify whether a variable is a confounder or not, as this depends on the specific study, and is consistent with previous publications [9, 83]. The use of a clinical expert review involving a questionnaire followed by individual interviews provides several advantages, particularly in its mixed methods research design that combines both quantitative and qualitative approaches. By doing so, the responses can be consolidated, while still offering an in‐depth understanding of the clinical experts' perspectives. Moreover, the inclusion of clinical experts from different countries ensures a diverse range of clinical experiences. While invaluable, it should be noted that conducting a clinical review by experts following an SLR can lengthen timelines considerably, which underscores the need to plan in advance and conduct the review in close proximity to the SLR.

This study has certain limitations that should be noted when interpreting the results. First, the demographics, clinical and treatment characteristics, as well as treatment received by patients during the study period varied across the included studies. Although considered a strength of real‐world studies, the presence of heterogeneity may complicate interpretations of prognostic association estimates. Additionally, since most of the studies predominantly involved White or Asian populations when reported, the study findings may not be generalizable across all racial and ethnic subgroups. Furthermore, only variables and clinical outcomes with statistically significant associations (*p* < 0.05) were extracted. Given that statistical significance is highly influenced by sample and effect size, this study may not include an exhaustive list of every prognostic factor or EMM relevant to the patient population. In addition, there are several instances in which only one study reported a significant prognostic factor‐clinical outcome association, limiting the reliability of conclusions drawn regarding such associations. Most of the included studies had a high or moderate risk of bias due to lack of reporting, specifically in the “Study Attrition” and “Statistical Analysis and Reporting” domains. This may be due to insufficient reporting, particularly in conference abstracts, and the fact that many prognostic factor analyses were exploratory in nature and not typically the primary objective of the included studies. Finally, although we recognize the importance of the pivotal trials of CAR T‐cell therapies [84, 85, 86] and polatuzumab vedotin [87] in shaping the current standard of care for R/R DLBCL, these trials were excluded from our review based on pre‐specified methodological criteria, as no statistically significant prognostic factor or EMM was reported in these studies. The exclusion of these pivotal studies may affect the generalizability of our findings and highlight a gap in our understanding of the prognostic factors and EMMs among patients treated with these established therapies based on clinical trial evidence. However, multiple studies reporting prognostic factors in patients treated with CAR T‐cell therapies or polatuzumab vedotin in real‐world practice have been identified and included in this SLR, reflecting the current standard of care. With the growing role of CAR T‐cell therapy in the treatment landscape, and the increasing availability of data on patients for whom it fails, previous use of CAR T‐cell therapy may become an important factor to consider for the prognosis of patients with R/R DLBCL, especially for those receiving later LoTs.

## 5 Conclusions

SLR‐based a priori identification of prognostic factors combined with expert clinical review provides a robust multi‐method approach to evaluating and ranking the level of evidence to assist in selecting prognostic factors for pre‐specified comparative analyses, such as those between single‐arm trials and real‐world cohorts.

## Author Contributions

B.v.T., P.A., I.Z., Á.S., Y.K., J.U., M.S., L.W., E.T., K.C., E.R., C.H., J.J.J., A.N.A., Y.X., Sh.A., Sr.A., H.M., Q.M., and A.J.U. made substantial contributions to the conception or design of the work; or the acquisition, analysis, or interpretation of data for the work. M.S. wrote the manuscript. All authors reviewed the work critically for important intellectual content. All authors provided final approval of the version to be published and agreed to be accountable for all aspects of the work in ensuring that questions related to the accuracy or integrity of any part of the work are appropriately investigated and resolved.

## Ethics Statement

This systematic literature review did not require ethics approval or consent to participate. The information used in this review was reported in published articles and reported in the aggregate.

## Conflicts of Interest

B.v.T. is an advisor or consultant for Allogene, Amgen, BMS/Celgene, Cerus, Gilead Kite, Incyte, IQVIA, Janssen‐Cilag, Lilly, Merck Sharp & Dohme, Miltenyi, Novartis, Noscendo, Pentixapharm, Pfizer, Pierre Fabre, Qualworld, Regeneron, Roche, Serb, Sobi, and Takeda; has received honoraria from AbbVie, AstraZeneca, BMS/Celgene, Gilead Kite, Incyte, Janssen‐Cilag, Lilly, Merck Sharp & Dohme, Novartis, Roche, Serb, and Takeda; reports research funding from Esteve (Inst), Merck Sharp & Dohme (Inst), Novartis (Inst), and Takeda (Inst); reports travel support from AbbVie, AstraZeneca, Gilead Kite, Janssen‐Cilag, Lilly, Merck Sharp & Dohme, Novartis, Pierre Fabre, Roche, and Takeda; and is a member of steering committees for Regeneron (Inst) and Takeda. P.A. has received honoraria from AbbVie, AstraZeneca, BeiGene, BMS, Genmab, Incyte, Janssen, Regeneron, and Roche. I.Z. and Á.S.P report no conflicts of interest. Y.K., J.U., L.W., E.T., K.C., and E.R. are employees of IQVIA Inc. M.S. was an employee of IQVIA Inc. when the study was conducted, was supported by the National Institutes of General Medical Sciences grant T32GM‐075766 from 2019 to 2022, while at the University of Pennsylvania Perelman School of Medicine, and received an International Society for Pharmacoepidemiology Grant Scholarship in 2022. The funding bodies had no role in the design and conduct of this study; collection, management, analysis, and interpretation of the data; preparation, review, or approval of the abstract; or the decision to submit for publication. C.H., J.J.J., A.N.A., Y.X., H.M., and Q.M. are employees of Regeneron Pharmaceuticals Inc. Sr. A. was an employee of Regeneron Pharmaceuticals Inc. when the study was conducted as is a current employee of Beam Therapeutics. Sh.A. was an employee of Regeneron Pharmaceuticals Inc. when the study was conducted and is a current employee of Landmark Science Inc. A.J.U. has received honoraria from AbbVie, Eli Lilly, Gilead Kite, Incyte, Janssen, Regeneron, Roche, and Sandoz.

## Supporting information


**Data S1.** Supporting Information.

## Data Availability

For full transparency, we have made available in the manuscript and Supporting Information S1: Appendix: the search strategy, PRISMA flow diagram with reasons for exclusion, study level risk of bias, full list of references of included studies, table of study characteristics, and table of outcome data.

## References

[1] National Comprehensive Cancer Network , “NCCN Guidelines for B‐Cell Lymphomas,” Version 3.2024, May 2024, https://www.nccn.org/guidelines/guidelines‐detail?category=1&id=1480.

[2] M. Ernst , U. Dührsen , D. Hellwig , G. Lenz , N. Skoetz , and P. Borchmann , “Diffuse Large B‐Cell Lymphoma and Related Entities,” Deutsches Ärzteblatt International 120, no. 17 (2023): 289–296. 36942797 10.3238/arztebl.m2023.0035 PMC10391525

[3] M. Federico , S. Luminari , A. Dondi , et al., “R‐CVP Versus R‐CHOP Versus R‐FM for the Initial Treatment of Patients With Advanced‐Stage Follicular Lymphoma: Results of the FOLL05 Trial Conducted by the Fondazione Italiana Linfomi,” Journal of Clinical Oncology 31, no. 12 (2013): 1506–1513. 23530110 10.1200/JCO.2012.45.0866

[4] J. W. Friedberg , “Relapsed/Refractory Diffuse Large B‐Cell Lymphoma,” Hematology. American Society of Hematology. Education Program 2011 (2011): 498–505. 22160081 10.1182/asheducation-2011.1.498

[5] L. H. Sehn and G. Salles , “Diffuse Large B‐Cell Lymphoma,” New England Journal of Medicine 384, no. 9 (2021): 842–858. 33657296 10.1056/NEJMra2027612 PMC8377611

[6] S. El Warrak , M. A. Kharfan‐Dabaja , M. Iqbal , M. Hamadani , J. Chavez , and R. Mohty , “Therapeutic Options for Large B‐Cell Lymphoma Relapsing After CD19‐Directed CAR T‐Cell Therapy,” Bone Marrow Transplantation 59, no. 2 (2024): 162–170. 38102213 10.1038/s41409-023-02176-0

[7] M. Crump , S. S. Neelapu , U. Farooq , et al., “Outcomes in Refractory Diffuse Large B‐Cell Lymphoma: Results From the International SCHOLAR‐1 Study,” Blood 130, no. 16 (2017): 1800–1808. 28774879 10.1182/blood-2017-03-769620 PMC5649550

[8] P. L. Zinzani , T. Rodgers , D. Marino , et al., “RE‐MIND: Comparing Tafasitamab + Lenalidomide (L‐MIND) With a Real‐World Lenalidomide Monotherapy Cohort in Relapsed or Refractory Diffuse Large B‐Cell Lymphoma,” Clinical Cancer Research 27, no. 22 (2021): 6124–6134. 34433649 10.1158/1078-0432.CCR-21-1471 PMC9414300

[9] H. Van Le , K. Van Naarden Braun , G. S. Nowakowski , et al., “Use of a Real‐World Synthetic Control Arm for Direct Comparison of Lisocabtagene Maraleucel and Conventional Therapy in Relapsed/Refractory Large B‐Cell Lymphoma,” Leukemia & Lymphoma 64, no. 3 (2023): 573–585. 36755418 10.1080/10428194.2022.2160200

[10] Institute for Quality and Efficiency in Health Care (IQWiG) , “General Methods (Version 6.0 of 5 November 2020),” 2020, https://www.iqwig.de/methoden/general‐methods_version‐6‐0.pdf. 27403465

[11] J. P. T. Higgins , J. Thomas , J. Chandler , et al., “Cochrane Handbook for Systematic Reviews of Interventions. Cochrane; Version 6.2 (Updated February 2021),” 2021, https://training.cochrane.org/handbook/archive/v6.2.

[12] M. J. Page , J. E. McKenzie , P. M. Bossuyt , et al., “The PRISMA 2020 Statement: An Updated Guideline for Reporting Systematic Reviews,” Journal of Clinical Epidemiology 134 (2021): 178–189. 33789819 10.1016/j.jclinepi.2021.03.001

[13] The European Network of Centres for Pharmacoepidemiology and Pharmacovigilance (ENCePP) , “Guide on Methodological Standards in Pharmacoepidemiology (Revision 11),” 2023, https://encepp.europa.eu/encepp‐toolkit/methodological‐guide_en.

[14] U.S. Food and Drug Administration (FDA) , “Meta‐Analyses of Randomized Controlled Clinical Trials to Evaluate the Safety of Human Drugs or Biological Products,” 2018, https://www.fda.gov/regulatory‐information/search‐fda‐guidance‐documents/meta‐analyses‐randomized‐controlled‐clinical‐trials‐evaluate‐safety‐human‐drugs‐or‐biological.

[15] National Institute for Health and Care Excellence (NICE) , “Guide to the Methods of Technology Appraisal,” 2013, https://www.nice.org.uk/process/pmg9/resources/guide‐to‐the‐methods‐of‐technology‐appraisal‐2013‐pdf‐2007975843781. 27905712

[16] National Institute for Health and Care Excellence (NICE) , “Single Technology Appraisal and Highly Specialised Technologies Evaluation: User Guide for Company Evidence Submission Template,” 2015, https://www.nice.org.uk/process/pmg24/resources/single‐technology‐appraisal‐and‐highly‐specialised‐technologies‐evaluation‐user‐guide‐for‐company‐evidence‐submission‐template‐pdf‐72286715419333.

[17] M. Ernst , A. Oeser , B. Besiroglu , et al., “Chimeric Antigen Receptor (CAR) T‐Cell Therapy for People With Relapsed or Refractory Diffuse Large B‐Cell Lymphoma,” Cochrane Database of Systematic Reviews 9, no. 9 (2021): CD013365. 34515338 10.1002/14651858.CD013365.pub2 PMC8436585

[18] S. I. Khan , M. Y. Anwar , A. Rafae , et al., “Efficacy and Safety of Phosphoinositide 3‐Kinase (PI3K) Inhibitors in Non‐Hodgkin's Lymphoma: A Systematic Review and Meta‐Analysis,” Blood 136, no. Suppl 1 (2020): S12–S13, 10.1182/blood-2020-134986.

[19] L. Wang , H. Lam , Y. Shou , and A. Galaznik , “Meta‐Analytical Methods for Estimating Outcomes From Overall Response Rate in Patients With Relapsed/Refractory Diffuse Large B‐Cell Lymphoma,” Oncotarget 10, no. 35 (2019): 3285–3293. 31143374 10.18632/oncotarget.26904 PMC6524929

[20] A. Galaznik , J. A. Bell , M. M. Hoog , et al., “Systematic Review of Therapy Used in Relapsed or Refractory Diffuse Large B‐Cell Lymphoma,” Blood 128, no. 22 (2016): 3562. 10.4155/fsoa-2018-0049 PMC6088264 30112190

[21] H. Tilly , M. Gomes da Silva , U. Vitolo , et al., “Diffuse Large B‐Cell Lymphoma (DLBCL): ESMO Clinical Practice Guidelines for Diagnosis, Treatment and Follow‐Up,” Annals of Oncology 26, no. Suppl 5 (2015): v116–v125. 26314773 10.1093/annonc/mdv304

[22] A. D. Zelenetz , L. I. Gordon , J. E. Chang , et al., “NCCN Guidelines® Insights: B‐Cell Lymphomas, Version 5.2021,” Journal of the National Comprehensive Cancer Network 19, no. 11 (2021): 1218–1230. 34781267 10.6004/jnccn.2021.0054

[23] J. A. Hayden , D. A. van der Windt , J. L. Cartwright , P. Côté , and C. Bombardier , “Assessing Bias in Studies of Prognostic Factors,” Annals of Internal Medicine 158, no. 4 (2013): 280–286. 23420236 10.7326/0003-4819-158-4-201302190-00009

[24] R. O. Casasnovas , G. Follows , J. M. Zijlstra , et al., “Comparison of the Effectiveness and Safety of the Oral Selective Inhibitor of Nuclear Export, Selinexor, in Diffuse Large B Cell Lymphoma Subtypes,” Clinical Lymphoma, Myeloma & Leukemia 22, no. 1 (2022): 24–33. 10.1016/j.clml.2021.07.017 34493477

[25] M. Schuster , M. Canales , J. Westin , et al., “Lymphocyte Count Effect on Efficacy and Safety of Single Agent Oral Selinexor in Patients With Relapsed/Refractory Diffuse Large B‐Cell Lymphoma (DLBCL): A Post‐Hoc Analysis From Phase 2B Sadal Study,” in European Hematology Association (EHA) 2021 Virtual Congress Abstract Book HemaSphere, 2021.

[26] M. W. Schuster , M. A. Canales , J. Westin , et al., “Effect of Age on the Efficacy and Safety of Single Agent Oral Selinexor in Patients With Relapsed/Refractory Diffuse Large B‐Cell Lymphoma (DLBCL): A Post‐Hoc Analysis of the Sadal Pivotal Study,” Blood 136, no. Suppl 1 (2020): S5–S6, 10.1182/blood-2020-137020.

[27] J. M. Zijlstra , G. Follows , R. O. Casasnovas , et al., “The Association Between Patient Characteristics and the Efficacy and Safety of Selinexor in Diffuse Large B‐Cell Lymphoma in the SADAL Study,” Cancers (Basel) 14, no. 3 (2022): 791. 35159058 10.3390/cancers14030791 PMC8834328

[28] J. Hu , X. Wang , M. Zhang , Q. Chen , and X. Zhang , “Combination of Decitabine and Modified DHAO Regimen: A Potential Salvage Regimen for Relapsed/Refractory Diffuse Large B‐Cell Lymphoma After Second‐Line Treatment Failure,” Hematological Oncology 39, no. Suppl 2 (2021): Abstract 325. 10.3389/fonc.2021.687374 PMC8253157 34222013

[29] J. M. Zijlstra , G. Follows , R. O. Casasnovas , et al., “Efficacy and Safety of Single Agent Oral Selinexor in Patients With Primary Refractory Diffuse Large B‐Cell Lymphoma (DLBCL): A Post‐Hoc Analysis of the SADAL Study,” in European Hematology Association (EHA) 2020 Virtual Congress Abstract Book HemaSphere, 2020.

[30] M. Maerevoet , J. Vermaat , M. A. Canales , et al., “Single Agent Oral Selinexor Demonstrates Deep and Durable Responses in Relapsed/Refractory Diffuse Large B‐Cell Lymphoma (DLBCL) in Both GCB and Non‐GCB Subtypes: The Phase 2b Sadal Study,” Blood 132, no. Suppl 1 (2018): S1677, 10.1182/blood-2018-99-116868.

[31] S. A. Graf , R. D. Cassaday , K. Morris , et al., “Ibrutinib Monotherapy in Relapsed or Refractory, Transformed Diffuse Large B‐Cell Lymphoma,” Clinical Lymphoma, Myeloma & Leukemia 21, no. 3 (2021): 176–181. 10.1016/j.clml.2020.11.023 PMC7904587 33358575

[32] B. R. Búa , A. Jiménez‐Ubieto , J. J. Sánchez Blanco , et al., “ABCL‐181: Updated Results of a Phase 2 Study From GELTAMO Investigating the Combination of Ibrutinib With R‐GEMOX in Patients With Relapsed or Refractory Diffuse Large B‐Cell Lymphoma,” Clinical Lymphoma, Myeloma & Leukemia 21 (2021): S381.

[33] B. R. Búa , A. Jiménez‐Ubieto , J. J. Sánchez Blanco , et al., “Ibrutinib in Combination With R‐Gemox‐D in Patients With Relapsed or Refractory Diffuse Large B‐Cell Lymphoma: Phase II Clinical Trial of the Geltamo Group,” Blood 136, no. Suppl 1 (2020): S16–S17, 10.1182/blood-2020-140786.

[34] W. Sang , M. Shi , J. Yang , et al., “Phase II Trial of Co‐Administration of CD19‐ and CD20‐Targeted Chimeric Antigen Receptor T Cells for Relapsed and Refractory Diffuse Large B Cell Lymphoma,” Cancer Medicine 9, no. 16 (2020): 5827–5838. 32608579 10.1002/cam4.3259 PMC7433814

[35] S. A. Graf , R. D. Cassaday , K. K. Morris , et al., “Ibrutinib Is Effective in Relapsed or Refractory Transformed Indolent B‐Cell Non‐Hodgkin Lymphoma: Results From a Prospective Phase II Study,” Blood 132, no. Suppl 1 (2018): S2954, 10.1182/blood-2018-99-115476.

[36] D. Cohen , E. Luttwak , O. Beyar‐Katz , et al., “[^18^F] FDG PET‐CT in Patients With DLBCL Treated With CAR‐T Cell Therapy: A Practical Approach of Reporting Pre‐ and Post‐Treatment Studies,” European Journal of Nuclear Medicine and Molecular Imaging 49, no. 3 (2022): 953–962. 34480603 10.1007/s00259-021-05551-5

[37] Y.‐W. Wang , X. C. H. Tsai , H. A. Hou , et al., “Polatuzumab Vedotin‐Based Salvage Immunochemotherapy as Third‐Line or Beyond Treatment for Patients With Diffuse Large B‐Cell Lymphoma: A Real‐World Experience,” Annals of Hematology 101, no. 2 (2022): 349–358. 34766217 10.1007/s00277-021-04711-9

[38] A. Bajwa , Y. Huang , R. Li , et al., “Prognostic Value of Early Imaging Following CAR T‐Cell Therapy in DLBCL,” Journal of Clinical Oncology 39, no. 15 Suppl (2021): e19559, 10.1200/JCO.2021.39.15_suppl.e19559.

[39] A. Di Rocco , A. Cuneo , A. Di Rocco , et al., “Relapsed/Refractory Diffuse Large B‐Cell Lymphoma Patients. A Multicenter Retrospective Analysis of Eligibility Criteria for CAR‐T Cell Therapy,” Leukemia & Lymphoma 62, no. 4 (2021): 828–836. 33274677 10.1080/10428194.2020.1849676

[40] B. Eastman , D. S. Hippe , S. C. Smith , et al., “Pilot Prognostic Model for Survival in r/r DLBCL Patients Receiving Palliative Radiation Therapy,” International Journal of Radiation Oncology, Biology, Physics 111, no. 3 (2021): e299–e300, 10.1016/j.ijrobp.2021.07.943.

[41] S. Fried , R. Shouval , N. Varda‐Bloom , et al., “Patients With Out of Specification Tisagenlecleucel Can Be Salvaged With a Point‐of‐Care CAR T‐Cells: An Observational Intention‐to‐Treat Single‐Center Analysis,” Hematological Oncology 39, no. Suppl 2 (2021): Abstract 270.

[42] M. Hu , M. P. Watkins , Q. Cao , et al., “Predictors of Relapse and Survival Following Autologous Stem Cell Transplant in Patients With Diffuse Large B‐Cell Lymphoma,” Blood 138, no. Suppl 1 (2021): S1832, 10.1182/blood-2021-146734.

[43] M. Northend , W. Wilson , W. Osborne , et al., “Polatuzumab Vedotin With Bendamustine and Rituximab for Relapsed/Refractory High‐Grade B‐Cell Lymphoma: The UK Experience,” Hematological Oncology 39, no. Suppl 2 (2021): Abstract 174.

[44] Y. Segman , E. Ribakovsky , A. Avigdor , et al., “Outcome of Relapsed/Refractory Diffuse Large B‐Cell Lymphoma Patients Treated With Polatuzumab Vedotin‐Based Therapy: Real‐Life Experience,” Leukemia & Lymphoma 62, no. 1 (2021): 118–124. 32981410 10.1080/10428194.2020.1824069

[45] N. N. Shah , K. W. Ahn , C. Litovich , et al., “Is Autologous Transplant in Relapsed DLBCL Patients Achieving Only a PET+ PR Appropriate in the CAR T‐Cell Era?,” Blood 137, no. 10 (2021): 1416–1423. 33120429 10.1182/blood.2020007939 PMC7955408

[46] V. Buecklein , V. Blumenburg , J. Ackermann , et al., “Single‐Center Experience With Axicabtagene‐Ciloleucel (Axi‐Cel) and Tisagenlecleucel (Tisa‐Cel) for Relapsed/Refractory Diffuse Large B‐Cell Lymphoma: Comparable Response Rates and Manageable Toxicity,” Blood 136, no. Suppl 1 (2020): S34–S35, 10.1182/blood-2020-142932.

[47] D. Dujmovic , S. Basic‐Kinda , J. Sincic‐Petricevic , et al., “Polatuzumab‐Vedotin Combined With Immunochemotherapy in R/R Patients With DLBCL: A Retrospective, Non‐Interventional, Real‐Life Study of KROHEM, the Croatian Cooperative Group for Hematologic Diseases,” in European Hematology Association (EHA) 2020 Virtual Congress; Abstract Book; HemaSphere, 2020, 586.

[48] E. González‐Barca , A. Boumendil , D. Blaise , et al., “Outcome in Patients With Diffuse Large B‐Cell Lymphoma Who Relapse After Autologous Stem Cell Transplantation and Receive Active Therapy. A Retrospective Analysis of the Lymphoma Working Party of the European Society for Blood and Marrow Transplantation (EBMT),” Bone Marrow Transplantation 55, no. 2 (2020): 393–399. 31541205 10.1038/s41409-019-0650-x

[49] C.‐H. Tsai , F. M. Tien , H. A. Hou , et al., “Polatuzumab Vedotin‐Based Salvage Chemotherapy in the Third‐Line or Above Treatment for Diffuse Large B‐Cell Lymphoma,” Blood 136, no. Suppl 1 (2020): S12, 10.1182/blood-2020-138481.

[50] I. Khouri , D. Milton , Y. Wang , et al., “Clinical Relevance of MYC/BCL2, Cell of Origin and Conditioning Regimen in Patients With Relapsed Diffuse Large B‐Cell Lymphoma (DLBCL) Treated With Allogeneic Stem Cell Transplantation (alloSCT),” in European Hematology Association (EHA) 2020 Virtual Congress Abstract Book HemaSphere, 2020.

[51] F. Manji and D. A. Stewart , “Real World Characteristics and Outcomes of Patients With Relapsed and Refractory Diffuse Large B Cell Lymphoma; a Provincial Experience,” Blood 136, no. Suppl 1 (2020): S17–S18.

[52] M. Mei , M. Hamadani , K. W. Ahn , et al., “Autologous Hematopoietic Cell Transplantation in Diffuse Large B‐Cell Lymphoma After Three or More Lines of Prior Therapy: Evidence of Durable Benefit,” Haematologica 107, no. 5 (2022): 1214–1217. 35112554 10.3324/haematol.2021.279999 PMC9052914

[53] C. Mesguich , A. Roch , E. Hindié , et al., “Prognostic Utility of Pre‐Transplantation [^18^F] Fluorodeoxyglucose Positron Emission Tomography/Computed Tomography in Patients With Diffuse Large B‐Cell Lymphoma Who Underwent Rituximab, Dexamethasone, High‐Dose Cytarabine, Carboplatin Salvage Chemotherapy,” British Journal of Haematology 188, no. 2 (2020): 268–271. 31388998 10.1111/bjh.16144

[54] D. Modi , S. Kim , M. Surapaneni , et al., “R‐BEAM Versus Reduced‐Intensity Conditioning Regimens in Patients Undergoing Allogeneic Stem Cell Transplantation for Relapsed Refractory Diffuse Large B Cell Lymphoma,” Biology of Blood and Marrow Transplantation 26, no. 4 (2020): 683–690. 31682979 10.1016/j.bbmt.2019.10.029 PMC7192775

[55] A. Nydegger , U. Bacher , M. N. Kronig , et al., “Analysis of Clinical and Laboratory Parameters Associated With Outcome After CAR‐T Treatment in DLBCL Patients,” in Swiss Hematology & Oncology Congress (SOHC), 2020.

[56] C. Quivoron , J. M. Michot , V. Camara‐Clayette , et al., “High Incidence of TP53 and Epigenetic Modifying Oncogenes Mutations in a Large Cohort of Patients Enrolled in Phase 1 Clinical Trials for Relapsed or Refractory Diffuse Large B‐Cell Lymphoma,” Blood 136, no. Suppl 1 (2020): S10–S11, 10.1182/blood-2020-136114.

[57] A. Di Rocco , A. Di Rocco , A. Farcomeni , et al., “Relapsed/Refractory Diffuse Large B‐Cell Lymphoma (R/R DLBCL) Patients: A Retrospective Analysis of Eligibility Criteria for CAR‐T Cell Therapy,” Blood 134, no. Suppl 1 (2019): S2888.

[58] M. J. Frank , N. Hossain , A. Bukhari , et al., “Detectable Circulating Tumor DNA 28 Days After the CD19 CAR T‐Cell Therapy, Axicabtagene Ciloleucel, Is Associated With Poor Outcomes in Patients With Diffuse Large B‐Cell Lymphoma,” Blood 134, no. Suppl 1 (2019): S884, 10.1182/blood-2019-132057.

[59] A. S. Kittai , A. J. Gordon , A. Mian , et al., “Comorbidities Predict Inferior Survival in Patients Receiving CAR T‐Cell Therapy for Relapsed/Refractory DLBCL: A Multicenter Retrospective Analysis,” Blood 134, no. Suppl 1 (2019): S780, 10.1182/blood-2019-124694.

[60] Y. Segman , E. Ribakovsky , A. Avigdor , et al., “Outcome of Relapsed DLBCL Patients, Treated With Polatuzumab‐BR or Polatuzumab‐R: Real Life Data,” Blood 134, no. Suppl 1 (2019): S5321.

[61] Y. Wang , K. H. Young , D. R. Milton , et al., “Clinical Relevance of MYC/BCL2 and Cell of Origin in Patients With Relapsed Diffuse Large B‐Cell Lymphoma Treated With Autologous Stem Cell Transplantation,” Blood 134, no. Suppl 1 (2019): S2021, 10.1182/blood-2019-126719.

[62] R. M. Myers , B. T. Hill , B. E. Shaw , et al., “Long‐Term Outcomes Among 2‐Year Survivors of Autologous Hematopoietic Cell Transplantation for Hodgkin and Diffuse Large B‐Cell Lymphoma,” Cancer 124, no. 4 (2018): 816–825. 29125192 10.1002/cncr.31114 PMC5871233

[63] R. Myers , B. T. Hill , B. E. Shaw , et al., “Long‐Term Outcomes Among Two‐Year Survivors of Autologous Hematopoietic Cell Transplant for Hodgkin and Diffuse Large B‐Cell Lymphoma,” Biology of Blood and Marrow Transplantation 23, no. 3 (2017): S27–S28.

[64] A. F. Herrera , M. Mei , L. Low , et al., “Relapsed or Refractory Double‐Expressor and Double‐Hit Lymphomas Have Inferior Progression‐Free Survival After Autologous Stem‐Cell Transplantation,” Journal of Clinical Oncology 35, no. 1 (2017): 24–31. 28034071 10.1200/JCO.2016.68.2740 PMC5455688

[65] L. S. Cerrada , R. Saliba , S. Srour , et al., “The Cell of Origin Has No Prognostic Impact on High‐Dose Chemotherapy With R‐Beam and Autologous Stem Cell Transplant for Diffuse Large B Cell Lymphoma,” Bone Marrow Transplantation 52, no. Suppl 1 (2017): S364–S365.

[66] E. Van Den Neste , N. Schmitz , N. Mounier , et al., “Outcomes of Diffuse Large B‐Cell Lymphoma Patients Relapsing After Autologous Stem Cell Transplantation: An Analysis of Patients Included in the CORAL Study,” Bone Marrow Transplantation 52, no. 2 (2017): 216–221. 27643872 10.1038/bmt.2016.213

[67] E. Van Den Neste , N. Schmitz , N. Mounier , et al., “Outcome of Patients With Relapsed Diffuse Large B‐Cell Lymphoma Who Fail Second‐Line Salvage Regimens in the International CORAL Study,” Bone Marrow Transplantation 51, no. 1 (2016): 51–57. 26367239 10.1038/bmt.2015.213

[68] T. A. Eyre , K. M. Linton , P. Rohman , et al., “Results of a Multicentre UK‐Wide Retrospective Study Evaluating the Efficacy of Pixantrone in Relapsed, Refractory Diffuse Large B Cell Lymphoma,” British Journal of Haematology 173, no. 6 (2016): 896–904. 26956150 10.1111/bjh.14021

[69] T. S. Fenske , K. W. Ahn , T. M. Graff , et al., “Allogeneic Transplantation Provides Durable Remission in a Subset of DLBCL Patients Relapsing After Autologous Transplantation,” British Journal of Haematology 174, no. 2 (2016): 235–248. 26989808 10.1111/bjh.14046 PMC4940282

[70] M. Gil‐Cupello , G. L. Shah , M. A. Maloy , et al., “Outcomes of Diffuse Large B‐Cell Lymphoma (DLBCL) Patients Progressing After Autologous Hematopoietic Stem Cell Transplant,” Biology of Blood and Marrow Transplantation 22, no. 3 (2016): S232–S233, 10.1016/j.bbmt.2015.11.641.

[71] A. G. Kettle , J. Switchenko , O. Calzada , et al., “Prolonged Progression‐Free Survival Is Possible in Patients With Diffuse Large B‐Cell Lymphoma Receiving > 1 Salvage Therapies Before Autologous Stem Cell Transplant,” Blood 128, no. 22 (2016): 5825.

[72] M. Maerevoet , J. M. Zijlstra , G. Follows , et al., “Survival Among Patients With Relapsed/Refractory Diffuse Large B Cell Lymphoma Treated With Single‐Agent Selinexor in the SADAL Study,” Journal of Hematology & Oncology 14, no. 1 (2021): 111. 34271963 10.1186/s13045-021-01122-1 PMC8283921

[73] J. Hu , X. Wang , F. Chen , et al., “Combination of Decitabine and a Modified Regimen of Cisplatin, Cytarabine and Dexamethasone: A Potential Salvage Regimen for Relapsed or Refractory Diffuse Large B‐Cell Lymphoma After Second‐Line Treatment Failure,” Frontiers in Oncology 11 (2021): 687374. 34222013 10.3389/fonc.2021.687374 PMC8253157

[74] N. Kalakonda , M. Maerevoet , F. Cavallo , et al., “Selinexor in Patients With Relapsed or Refractory Diffuse Large B‐Cell Lymphoma (SADAL): A Single‐Arm, Multinational, Multicentre, Open‐Label, Phase 2 Trial,” Lancet Haematology 7, no. 7 (2020): e511–e522. 32589977 10.1016/S2352-3026(20)30120-4

[75] S. P. Robinson , A. Boumendil , H. Finel , et al., “Autologous Stem Cell Transplantation for Relapsed/Refractory Diffuse Large B‐Cell Lymphoma: Efficacy in the Rituximab Era and Comparison to First Allogeneic Transplants. A Report From the EBMT Lymphoma Working Party,” Bone Marrow Transplantation 51, no. 3 (2016): 365–371. 26618550 10.1038/bmt.2015.286

[76] Z. Ying , L. Mi , N. Zhou , et al., “Prognostic Value of ^18^F‐Fluorodeoxyglucose Positron Emission Tomography Using Deauville Criteria in Diffuse Large B Cell Lymphoma Treated With Autologous Hematopoietic Stem Cell Transplantation,” Chinese Journal of Cancer Research 31, no. 1 (2019): 162–170, 10.21147/j.issn.1000-9604.2019.01.11. 30996574 PMC6433590

[77] Institute for Quality and Efficiency in Health Care (IQWiG) , “Rapid Report A19‐43: Concepts for the Generation of Routine Practice Data and Their Analysis for the Benefit Assessment of Drugs According to §35a Social Code Book V (SGB V) (Version 1.0; Status: 10 January 2020),” 2020, https://www.iqwig.de/download/a19‐43_routine‐practice‐data‐for‐the‐benefit‐assessment‐of‐drugs_rapid‐report_v1‐0.pdf.

[78] R. Vaidya and T. E. Witzig , “Prognostic Factors for Diffuse Large B‐Cell Lymphoma in the R(X)CHOP Era,” Annals of Oncology 25, no. 11 (2014): 2124–2133. 24625454 10.1093/annonc/mdu109 PMC4288137

[79] L. H. Sehn , “Paramount Prognostic Factors That Guide Therapeutic Strategies in Diffuse Large B‐Cell Lymphoma,” Hematology. American Society of Hematology. Education Program 2012, no. 1 (2012): 402–409. 23233611 10.1182/asheducation-2012.1.402

[80] International Non‐Hodgkin's Lymphoma Prognostic Factors Project , “A Predictive Model for Aggressive Non‐Hodgkin's Lymphoma,” New England Journal of Medicine 329, no. 14 (1993): 987–994. 8141877 10.1056/NEJM199309303291402

[81] L. H. Sehn , B. Berry , M. Chhanabhai , et al., “The Revised International Prognostic Index (R‐IPI) is a Better Predictor of Outcome Than the Standard IPI for Patients With Diffuse Large B‐Cell Lymphoma Treated With R‐CHOP,” Blood 109, no. 5 (2007): 1857–1861. 17105812 10.1182/blood-2006-08-038257

[82] Z. Zhou , L. H. Sehn , A. W. Rademaker , et al., “An Enhanced International Prognostic Index (NCCN‐IPI) for Patients With Diffuse Large B‐Cell Lymphoma Treated in the Rituximab Era,” Blood 123, no. 6 (2014): 837–842. 24264230 10.1182/blood-2013-09-524108 PMC5527396

[83] G. S. Nowakowski , D. H. Yoon , P. Mondello , et al., “RE‐MIND2: Comparative Effectiveness of Tafasitamab Plus Lenalidomide Versus Polatuzumab Vedotin/Bendamustine/Rituximab (Pola‐BR), CAR‐T Therapies, and Lenalidomide/Rituximab (R2) Based on Real‐World Data in Patients With Relapsed/Refractory Diffuse Large B‐Cell Lymphoma,” Annals of Hematology 102, no. 7 (2023): 1773–1787. 37171597 10.1007/s00277-023-05196-4 PMC10261238

[84] S. J. Schuster , M. R. Bishop , C. S. Tam , et al., “Tisagenlecleucel in Adult Relapsed or Refractory Diffuse Large B‐Cell Lymphoma,” New England Journal of Medicine 380, no. 1 (2019): 45–56. 30501490 10.1056/NEJMoa1804980

[85] S. S. Neelapu , F. L. Locke , N. L. Bartlett , et al., “Axicabtagene Ciloleucel CAR T‐Cell Therapy in Refractory Large B‐Cell Lymphoma,” New England Journal of Medicine 377, no. 26 (2017): 2531–2544. 29226797 10.1056/NEJMoa1707447 PMC5882485

[86] J. S. Abramson , M. L. Palomba , L. I. Gordon , et al., “Lisocabtagene Maraleucel for Patients With Relapsed or Refractory Large B‐Cell Lymphomas (TRANSCEND NHL 001): A Multicentre Seamless Design Study,” Lancet 396, no. 10254 (2020): 839–852. 32888407 10.1016/S0140-6736(20)31366-0

[87] L. H. Sehn , A. F. Herrera , C. R. Flowers , et al., “Polatuzumab Vedotin in Relapsed or Refractory Diffuse Large B‐Cell Lymphoma,” Journal of Clinical Oncology 38, no. 2 (2020): 155–165. 31693429 10.1200/JCO.19.00172 PMC7032881

