# Supplementary material for: Prognostic Factors and Effect Modifiers in Patients With Relapse or Refractory Diffuse Large B‐Cell Lymphoma After Two Lines of Therapy: A Systematic Literature and Expert Clinical Review
Source: Eur J Haematol. 2025 May 9;115(2):104–16. doi: 10.1111/ejh.14423 (PMC12224567; doi:10.1111/ejh.14423)
Supplement: Supplementary file 1 — Data S1. Supporting Information. [file EJH-115-104-s001.docx]

**SUPPLEMENTARY APPENDIX**

**Appendix A – Search strategy for OVID-based searches**

Database(s):

EBM Reviews – Cochrane Central Register of Controlled Trials, November 2021

Embase, 1974 to December 10, 2021

Ovid MEDLINE^®^ and In-Process, In-Data-Review & Other Non-Indexed Citations and Daily, 1946 to December 10, 2021

Date of search: December 13, 2021

| # | Searches | Results | Notes |
| --- | --- | --- | --- |
| 1 | ("large b cell lymphoma*" or dlbcl or "large bcell lymphoma*").ab,ti. or diffuse large B cell lymphoma/or Lymphoma, Large B-Cell, Diffuse/ | 68,753 | Population string – title/abstract/MeSH/Emtree field search  In adult patients with R/R DLBCL, 3L+ therapies |
| 2 | treatment failure/or (fail* or relapse* or refractory or resistan* or intolerant or progress).ab,ti. | 6,537,619 | Population string – title/abstract/MeSH/Emtree field search |
| 3 | prognosis/or prognostic assessment/or confounding factors, epidemiologic/or effect modifier, epidemiologic/or (prognos* or confound* or "effect modif*" or predict* or score or factor* or regress* or hazard or associat* or "effect measure modif*" or subgroup* or correlat*).ab,ti. | 24,484,657 | Outcome string – title/abstract/MeSH/Emtree field search |
| 4 | 1 and 2 and 3 | 12,724 |  |
| 5 | (line* or salvage* or rescue or prior).ab,ti. | 6,209,817 | Treatment line |
| 6 | (survival or respon* or remission or control or "time to next treatment" or progress* or outcome* or switch* or mortality or death).ab,ti. | 23,224,947 | Clinical outcomes |
| 7 | 4 and 5 and 6 | 5388 | Population + outcome + treatment line + clinical outcome strings |
| 8 | remove duplicates from 7 | 4125 | Deduped |
| 9 | limit 8 to yr="2016 -Current" | 2353 | 2016 to current |
| 10 | limit 9 to english language | 2304 | English language |
| 11 | 10 not (commentary or editorial or letter or "case report*" or "case stud*").pt. | 2269 | Removing publication types not of interest |

Abbreviations: 3L+ = third-line treatment setting and above; DLBCL = diffuse large B-cell lymphoma; MeSH = Medical Subject Headings; R/R = relapsed/refractory.

**Appendix B – Patient, intervention, comparison, outcome, time, and setting (PICOTS) criteria.**

| Criteria | Description |
| --- | --- |
| Populations | - Adult patients with R/R DLBCL after two LoTs (3L+)   Other applicable eligibility criteria:   - Lymphoma type: only include studies with patients with 100% DLBCL, or if results were stratified for DLBCL; exclude studies with mixed lymphoma types where results were not stratified; CNS lymphoma or PMBCL were not considered subtypes of DLBCL, and excluded based on clinician input - LoT: include studies where at least 50% of patients received 3L+ therapy (i.e., median or mean of at least two prior LoTs); exclude studies that did not report the number of prior LoTs |
| Interventions | - Any or none |
| Comparators | - Not applicable |
| Outcomes^†^ | - Potential prognostic factors^‡^ or effect measure modifiers^§^ that were associated with objective response rate, overall survival, progression-free survival, time to next treatment, complete response rate, duration of response, or disease control rate |
| Time | - Publication date limit: January 1, 2016, to December 13, 2021 |
| Study design | - Include: randomized controlled trials, non-randomized controlled trials, observational studies - Exclude: case reports, evidence synthesis studies or reviews (flag for bibliography), health economic modeling/economic/resource use studies |
| Other | - Exclude: non-human, pediatric/pregnancy; publication type as editorials, letters, notes, commentaries - Geography: global - Language: English (journal article or conference abstract) |

Abbreviations: 3L+ = third-line treatment setting and above; CNS = central nervous system; DLBCL = diffuse large B-cell lymphoma; LoT = line of therapy; PMBCL = primary mediastinal large B-cell lymphoma; R/R = relapsed/refractory.

^†^Notes for outcomes: 1) The search and screening were kept broad in order to capture studies reporting on prognostic factors, predictive factors, correlation, association, confounders, effect measure modifiers, subgroups, and other related concepts. 2) Information was extracted for the statistically significant variables only. If multiple models were reported in a study, results were extracted from the most adjusted model. Studies were excluded if statistical significance was not concluded for any model variables.

^‡^Defined as variables, including confounders, that are associated with subsequent health outcomes among individuals with a particular health condition.

^§^Defined as factors that modify the effect of the putative causal factor(s) under study; effect measure modification occurs when the magnitude of the effect differs depending on the level of a third variable.

**Appendix C – Quality assessment tool**

Risk of bias assessment of individual studies was performed using the quality in prognostic studies (QUIPS) tool [1].

| Variable | Bias domains | | | | | |
| --- | --- | --- | --- | --- | --- | --- |
|  | 1. Study participants | 2. Study attrition | 3. Prognostic factor (PF) measurement | 4. Outcome measurement | 5. Study confounding | 6. Statistical analysis and reporting |
| **Optimal study or characteristics of unbiased study** | The study sample adequately represents the population of interest | The study data available (i.e., participants not lost to follow-up) adequately represent the study sample | The PF is measured in a similar way for all participants | The outcome of interest is measured in a similar way for all participants | Important potential confounding factors are appropriately accounted for | The statistical analysis is appropriate, and all primary outcomes are reported |
| **Prompting items and considerations** | a. Adequate participation in the study by eligible persons | a. Adequate response rate for study participants | a. A clear definition or description of the PF is provided | a. A clear definition of the outcome is provided | a. All important confounders are measured | a. Sufficient presentation of data to assess the adequacy of the analytic strategy |
|  | b. Description of the source population or population of interest | b. Description of attempts to collect information on participants who dropped out | b. Method of PF measurement is adequately valid and reliable | b. Method of outcome measurement used is adequately valid and reliable | b. Clear definitions of the important confounders measured are provided | b. Strategy for model building is appropriate and is based on a conceptual framework or mode |
|  | c. Description of the baseline study sample | c. Reasons for loss to follow-up are provided | c. Continuous variables are reported or appropriate cut points are used | c. The method and setting of outcome measurement are the same for all study participants | c. Measurement of all important confounders is adequately valid and reliable | c. The selected statistical model is adequate for the design of the study |
|  | d. Adequate description of the sampling frame and recruitment | d. Adequate description of participants lost to follow-up | d. The method and setting of measurement of PF are the same for all study participants |  | d. The method and setting of confounding measurement are the same for all study participants | d. There is no selective reporting of results |
|  | e. Adequate description of the period and place of recruitment | e. There are no important differences between participants who completed the study and those who did not | e. Adequate proportion of the study sample has complete data for the PF |  | e. Appropriate methods are used if imputation is used for missing confounder data |  |
|  | f. Adequate description of inclusion and exclusion criteria |  | f. Appropriate methods of imputation are used for missing PF data |  | f. Important potential confounders are accounted for in the study design |  |
|  |  |  |  |  | g. Important potential confounders are accounted for in the analysis |  |

| Ratings |  |  |  |  |  |  |
| --- | --- | --- | --- | --- | --- | --- |
| High risk of bias | The relationship between the PF and outcome is very likely to be different for participants and eligible nonparticipants | The relationship between the PF and outcome is very likely to be different for completing and noncompleting participants | The measurement of the PF is very likely to be different for different levels of the outcome of interest | The measurement of the outcome is very likely to be different related to the baseline level of the PF | The observed effect of the PF on the outcome is very likely to be distorted by another factor related to PF and outcome | The reported results are very likely to be spurious or biased related to analysis or reporting |
| Moderate risk of bias | The relationship between the PF and outcome may be different for participants and eligible nonparticipants | The relationship between the PF and outcome may be different for completing and noncompleting participants | The measurement of the PF may be different for different levels of the outcome of interest | The measurement of the outcome may be different related to the baseline level of the PF | The observed effect of the PF on outcome may be distorted by another factor related to PF and outcome | The reported results may be spurious or biased related to analysis or reporting |
| Low risk of bias | The relationship between the PF and outcome is unlikely to be different for participants and eligible nonparticipants | The relationship between the PF and outcome is unlikely to be different for completing and noncompleting participants | The measurement of the PF is unlikely to be different for different levels of the outcome of interest | The measurement of the outcome is unlikely to be different related to the baseline level of the PF | The observed effect of the PF on outcome is unlikely to be distorted by another factor related to PF and outcome | The reported results are unlikely to be spurious or biased related to analysis or reporting |

**Appendix D – Online questionnaire completed by the clinical experts**

Note: Questions 15–25 were related to follicular lymphoma (FL) Grade 1–3a, which is not a topic for this manuscript; therefore, only the questions related to diffuse large B-cell lymphoma (DLBCL) are presented in this appendix.


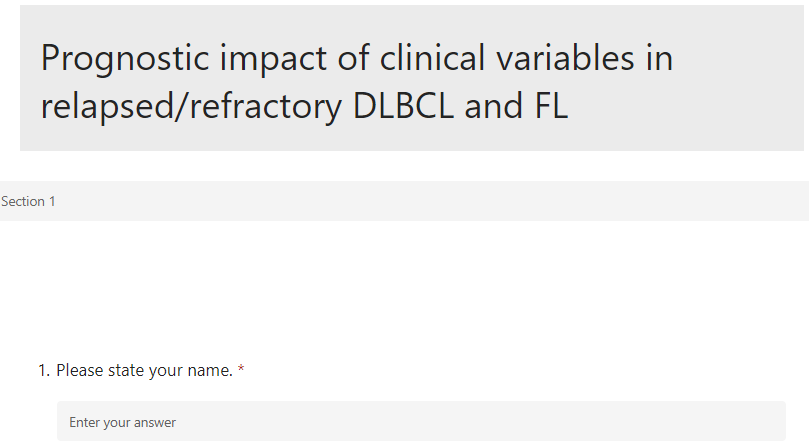


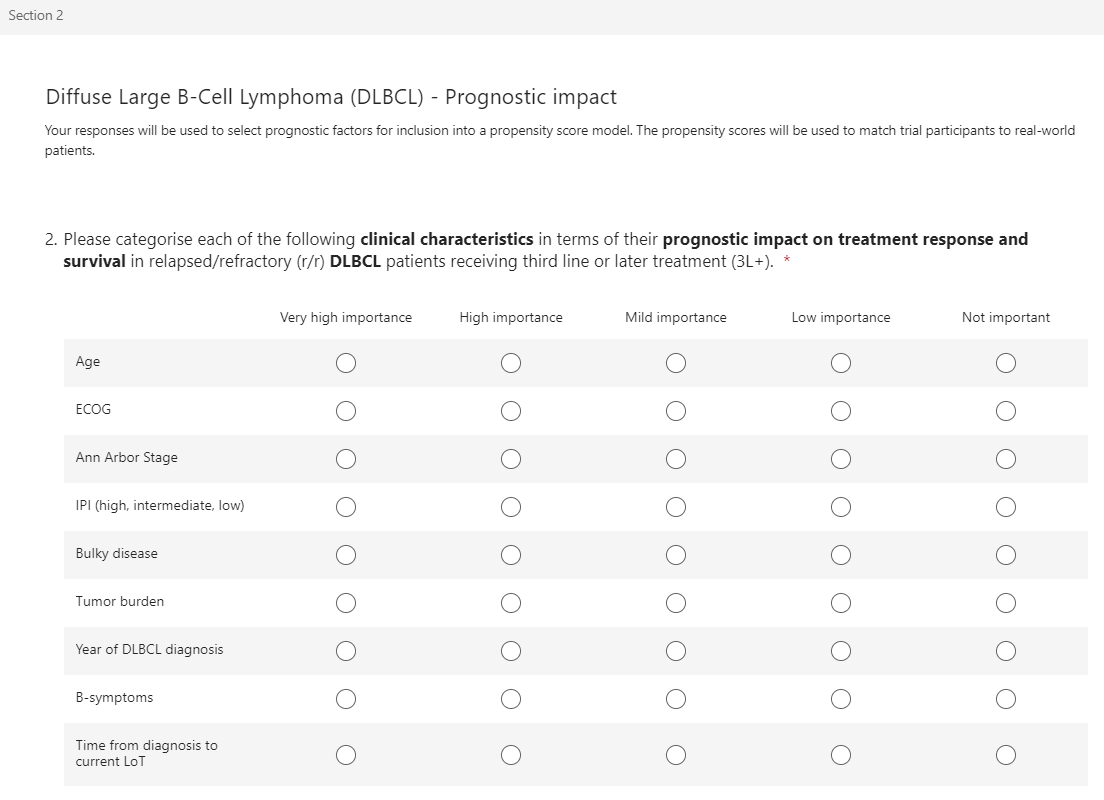


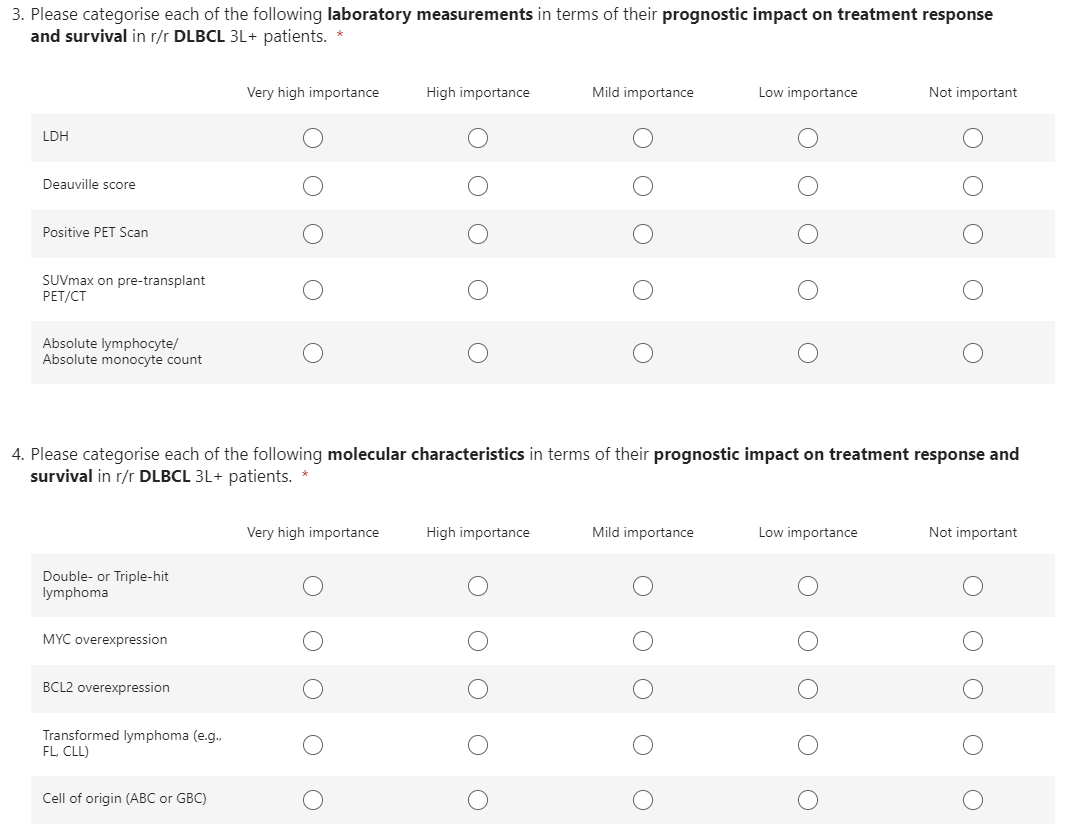


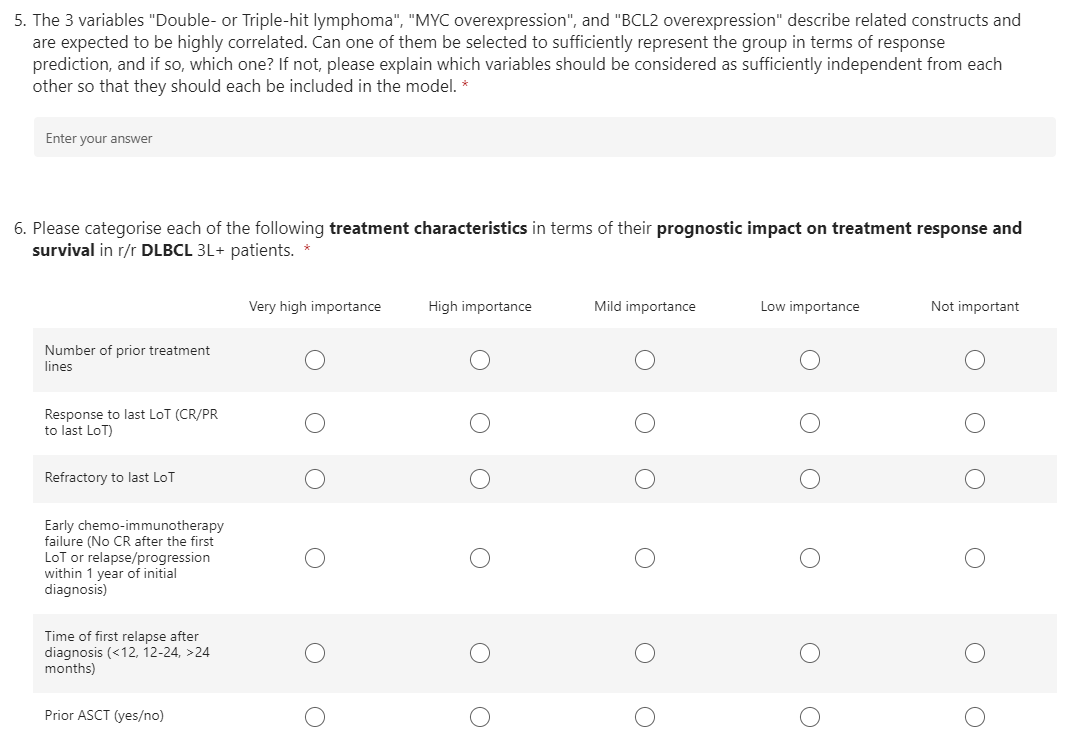


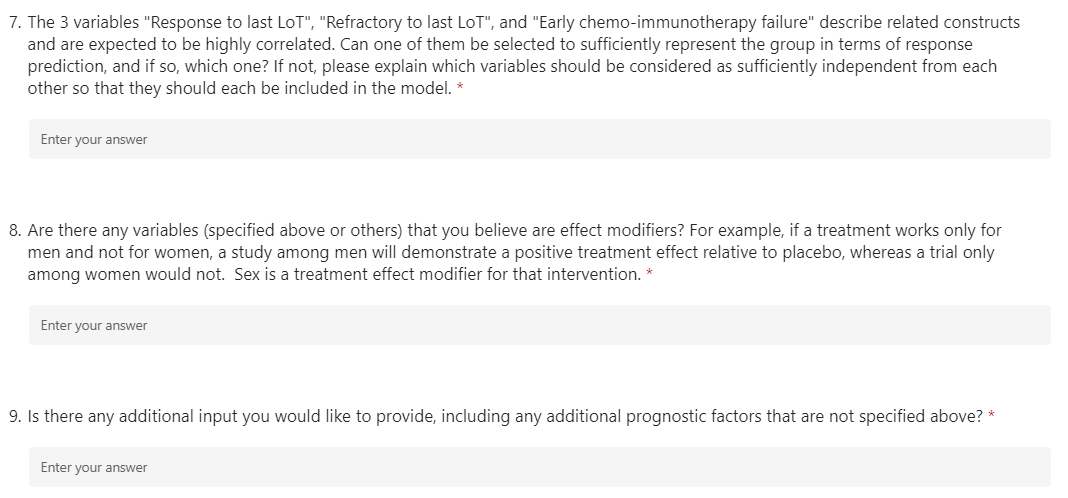


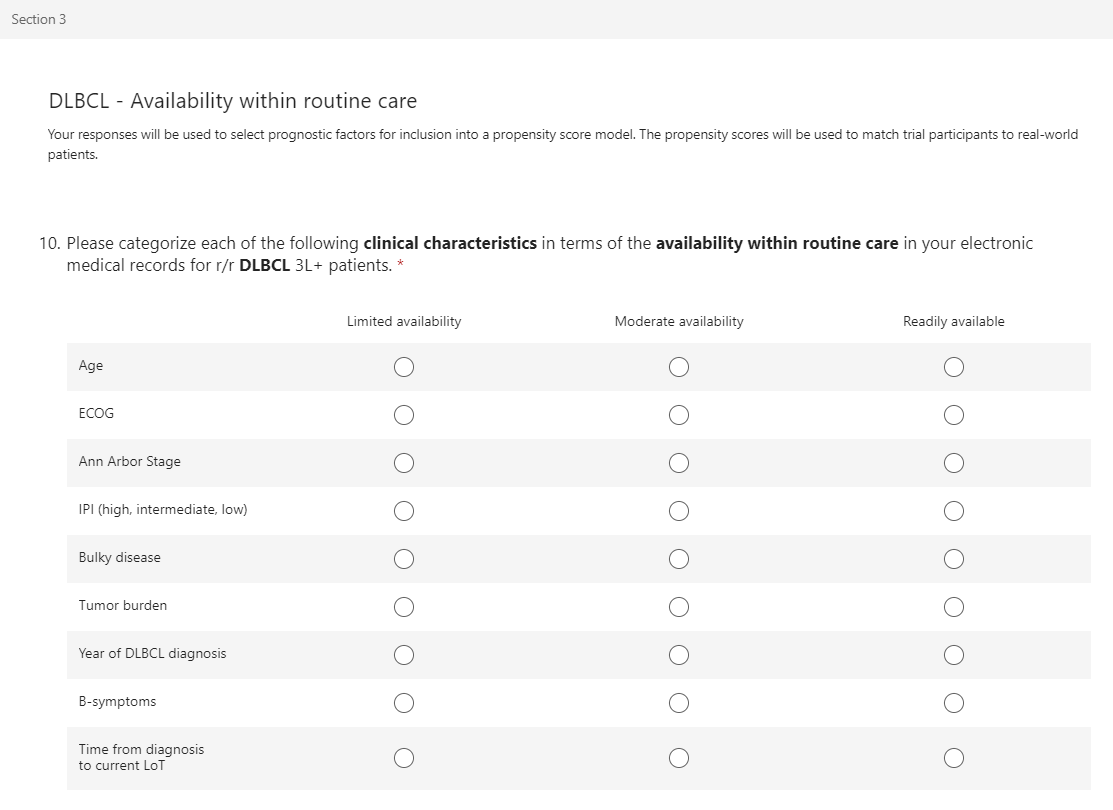


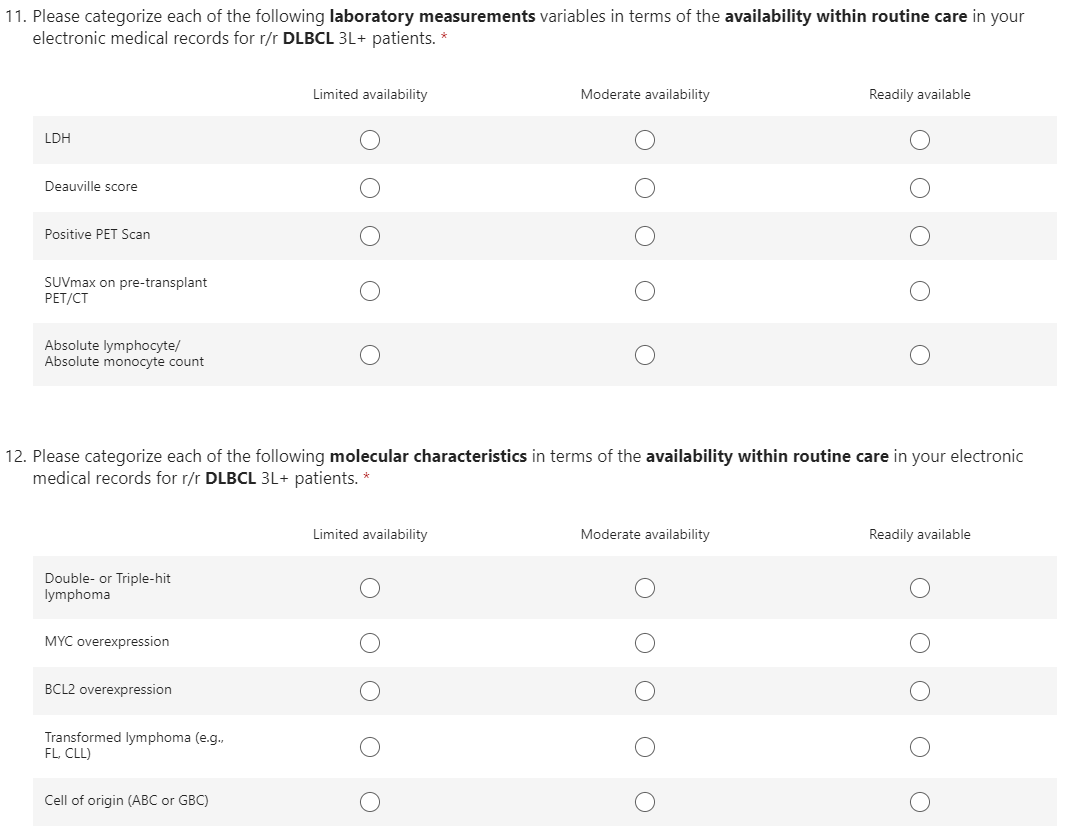


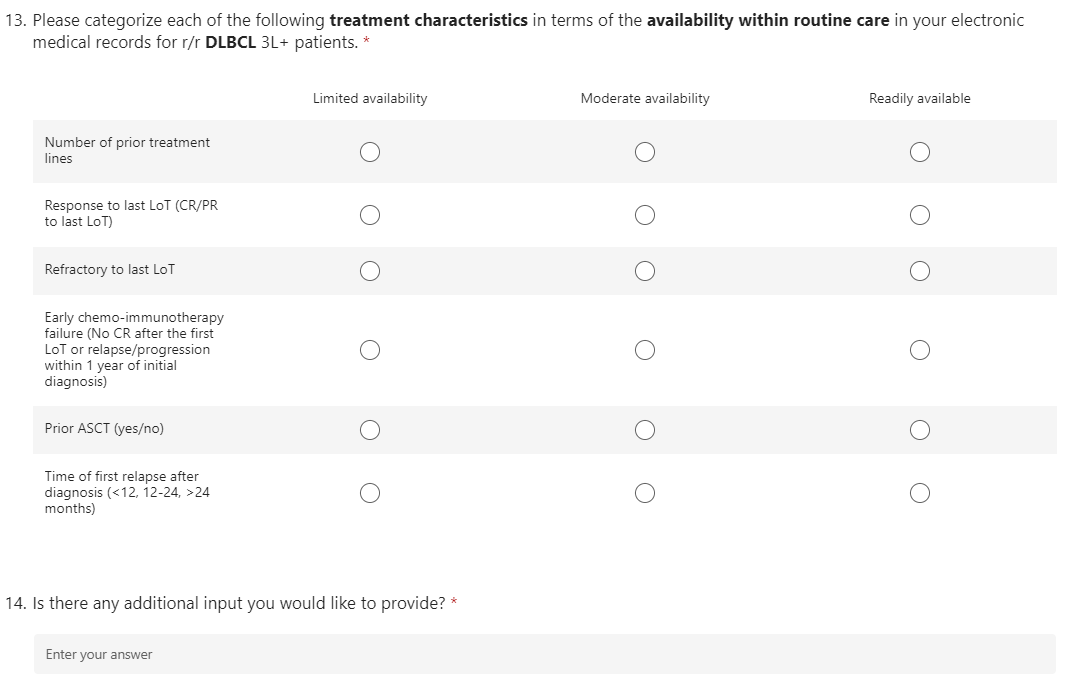


**Appendix E – Supplementary data for systematic literature review**

**TABLE S1** Study characteristics.

| Author, year  Study name; trial # | Study country | Study period | Median follow-up (range) | N for DLBCL | Intervention/ comparator | Source of population | Population description | Analytical approach | Median line of  prior therapy  (range) | Median age  (range) |
| --- | --- | --- | --- | --- | --- | --- | --- | --- | --- | --- |
| ***RCT*** |  |  |  |  |  |  |  |  |  |  |
| Casasnovas 2022 [2]  SADAL; NCT02227251^†^ | Australia, Austria, Belgium, Bulgaria, Canada, France, Germany, Greece, Hungary, India, Israel, Italy, Netherlands, New Zealand, Poland, Serbia, Spain, UK, US | November 2014 to August 2022 (final data collection date for primary outcome measure) | NR | 134 | Oral selinexor | The SADAL study | Patients with de novo DLBCL or DLBCL transformed from previously diagnosed indolent lymphoma | Log-rank test and Cox proportional hazards model | 2 (2–5) | GCB: 67 (44–  91)  Non-GCB:  69.5 (35–87)  De novo: 67  (35–91)  Transformed:  69 (48–82)  Double  expressors:  65 (47–86)  Nondouble  expressors:  69.5 (35–87) |
| Maerevoet 2021 [3] |  |  |  |  |  |  |  |  |  |  |
| Schuster 2021 [4]^‡^ |  |  |  |  |  |  |  |  |  |  |
| Schuster 2020 [5]^‡^ |  |  |  |  |  |  |  |  |  |  |
| Zijlstra 2022 [6]  SADAL; NCT02227251^†^ | Australia, Austria, Belgium, Bulgaria, Canada, France, Germany, Greece, Hungary, India, Israel, Italy, Netherlands, New Zealand, Poland, Serbia, Spain, UK, US | November 2014 to August 2022 (final data collection date for primary outcome measure) | NR | 134 | Oral selinexor | The SADAL study | Patients with DLBCL | The chi-squared test was used to compare proportions between subgroups. For time-to-event variables, the Kaplan-Meier method was used for descriptive summaries. Log-rank test and Cox proportional hazards model were used to compare survival distributions between subgroups Statistical analyses were performed using SAS | 2 (2–5) | 67 (35–91) |
| Hu 2021 [7]  NCT03579082 | China | June 1, 2018 to June 1, 2019 (final data collection date for primary outcome measure) | Median follow-up: 7.5 months (1.5–28 months) | 20 | R±DHAP + decitabine/no intervention: Arm B, R±DHAP | Hospital of Zhengzhou University | Patients with R/R DLBCL who failed second-line treatment | Prognostic risk factors and the 95% CI were estimated with univariate analysis. Statistical significance was defined as *p*< 0.05 | Number of prior  lines of therapy:  2: 14 (70%)  >2: 6 (30%) | 50.5 (30–65) |
| Hu 2021 [8]^‡^ |  |  |  |  |  |  |  |  |  |  |
| Kalakonda 2020 [9]  SADAL; NCT02227251^†^ | Australia, Austria, Belgium, Bulgaria, Canada, France, Germany, Greece, Hungary, India, Israel, Italy, Netherlands, New Zealand, Poland, Serbia, Spain, UK, US | November 2014 to August 2022 (final data collection date for primary outcome measure) | Median follow-up: 11.1 months (IQR 1.87–10.27) | 127 | Oral selinexor | The SADAL study | Patients with R/R DLBCL treated with single‑agent selinexor | Two-proportion z-test was used to test two group proportions | 2 (IQR 2.0–3.0) | 67 (35–87) |
| Zijlstra 2020 [10]^‡^ |  |  |  |  |  |  |  |  |  |  |
| Maerevoet 2018 [11]^‡^  SADAL; NCT02227251^†^ | Australia, Austria, Belgium, Bulgaria, Canada, France, Germany, Greece, Hungary, India, Israel, Italy, Netherlands, New Zealand, Poland, Serbia, Spain, UK, US | November 2014 to August 2022 (final data collection date for primary outcome measure) | NR | 110 | Oral selinexor | The SADAL study | Patients with R/R DLBCL | NR | 3 (2–5) | 67 |
| ***Non-randomized trial*** | | | | | | | | | | |
| Graf 2021 [12]  NCT02207062^†^ | US | October 2014–March 2019 | Median follow-up: 11 months (range 2–47) | 20 | Oral ibrutinib | Trial | Patients with R/R transformed DLBCL | Clinical variables known to be correlated with outcomes of aggressive lymphoma were evaluated for association with efficacy in exploratory analyses | 4 (2–9) | 68 (35–89) |
| Búa 2021 [13]^‡^  GELTAMO; NCT02692248 | Spain | April 7, 2016–September 2020 | Median follow‐up: 29 months (range 0.37–48.9) | 64 | Ibrutinib-R-GEMOX-Dexa | The Geltamo study | Patients with non-GCB DLBCL, with R/R disease and non-candidates for ASCT | Multivariate analysis | **2 (range NR)^§^** | 67 (25–84) |
| Búa 2020 [14]^‡^ |  |  |  |  |  |  |  |  |  |  |
| Sang 2020 [15]  NCT03207178 | China | March 2017–July 2018 | Median follow-up: 6.6 months (range 0.3–16.4)  The end of follow-up was October 31, 2018 | 21 | Fludarabine, cyclophosphamide, ifosfamide | Blood Disease Institute of Xuzhou Medical University | Patients with R/R DLBCL | For continuous variables that conform to normal distribution, t test was used. For those that do not conform to normal distribution, Wilcoxon signed rank test was used for paired samples, and Mann-Whitney U test was used for independent samples. *p*- values <0.05 were considered significant | **3 (1–6)^§^** | 55 (23–72) |
| Graf 2018 [16]^‡^  NCT02207062^†^ | US | October 2014–November 1, 2023 | NR | 17 | Oral ibrutinib | Trial | DLBCL transforming from indolent histologies | NR | 4 (2–9) | 68 (36–89) |
| ***Observational*** | | | | | | | | | | |
| Cohen 2022 [17] | Israel | April 2019–April 2021 | Median follow-up: 12.8 months (IQR 6.4–16.0) | 48 | CD19-specific CAR-T cell therapy | Medical records of Tel-Aviv Sourasky Medical Center | Patients with DLBCL treated with CAR-T cell therapy | Log-rank test and univariate Cox regression were applied to study the crude association between the studied predictors and OS and PFS  A multivariate Cox regression analysis was performed using a backward method (*p* > 0.1 was used as a criterion for removal) in order to identify independent predictors for OS. A two-sided *p*-value of <0.05 was considered statistically significant. Variables with a trend or a significant association to OS and PFS, as well as those known to be of important clinical significance, were tested in the multivariate model | 2 (2–4) | 68 (IQR 61–76) |
| Wang 2022 [18]  National Taiwan University Hospital 2018–2021^†^ | Taiwan | November 2018–April 2021 | Median follow-up: 18.8 months (range 0.3–30.2) | 40 | PoV | National Taiwan University Hospital, Taipei, Taiwan | Patients with R/R DLBCL (including de novo and transformed lymphoma) who had ever failed two or more lines of therapy and were treated with PoV-containing regimens | Continuous and discrete variables were compared using Kruskal–Wallis and chi-square tests, respectively. Time-to-event endpoints were evaluated by the Kaplan-Meier method, with differences between groups determined by the log-rank test. A two-tailed value of *p*< 0.05 was considered statistically significant | 4 (2–11) | 59 (20–82) |
| Bajwa 2021 [19]^‡^ | US | NR | NR | 111 | CAR-T cell therapies (tisa-cel and axi-cel) | The Ohio State University Comprehensive Cancer Center | Patients with R/R DLBCL treated with CAR-T cell therapy | Kaplan-Meier method and Cox model were used to correlate first scan result with OS | **3 (1–7)^§^** | 61 (23–84) |
| Di Rocco 2021 [20]  Four Italian centers 2010–2018^†^ | Italy | January 1, 2010–May 31, 2018 | NR | 137 | NR | Four Italian centers; retrospective database/clinical data | Patients with DLBCL with refractory disease or who have relapsed after ASCT | Cox proportional hazards model | 3 (2–8) | 63 (IQR 54.7–  71.2) |
| Eastman 2021 [21]^‡^ | US | 2009–2020 | Median follow-up: 6.3 months (range 0.1– 91.2) | 82 | NR | NR | Patients with R/R DLBCL who received their first course of pRT between 2009 and 2020 | A multivariable prognostic model was developed using Cox proportional hazards regression with LASSO-regularization, which optimizes variable inclusion based on the model’s overall prediction accuracy  Model discrimination performance was summarized using the concordance index after optimism-adjustment with the bootstrap (internal validation) | Number of prior  lines of therapy:  **≥3: 50%^§^** | Mean age: 62 |
| Fried 2021 [22]^‡^ | Israel | NR | Median follow-up: 14.7 months (IQR 13–17) | 37 | Tisa-cel | NR | Patients with DLBCL treated with tisa-cel | Multivariable ITT Cox regression | 2 (2–6) | 63 (28–79) |
| Hu 2021 [23] | US | 2010–2020 | Median follow-up: 62.4 months (5.2 years) from time of ASCT | 235 | ASCT | Medical records from University of Minnesota and Washington University School of Medicine | Adult patients with DLBCL who underwent ASCT | Univariate and multivariate Cox proportional hazards regression performed to identify factors associating with PFS and OS, summarized using HR with 95% CI | **2 (1–5)^§^** | 61 (25–75) |
| Northend 2021 [24]^‡^ | UK | July 2019–October 2020 | Median follow‐up: 7.7 months | 133 | Pola‐BR | 28 UK hospitals | Patients with DLBCL treated with Pola‐BR | Univariate and multivariate model | Number of prior  lines of therapy:  **≥2: 64.7%^§^** | 72 (18–88) |
| Segman 2021 [25]  Israeli medical centers 2018–2019^†^ | Israel | June 2018–September 2019 | Median follow-up: 6.8 months (range 0.6–14.8) | 47 | PoV | 14 Israeli medical centers | Patients with R/R DLBCL treated with PoV | The association between categorical variables and ORR was assessed by the chi-square and Fisher’s exact tests, and independent samples t tests and Mann–Whitney tests were applied to study the association between ORR and continuous variables  The association between each predictor and study outcomes was evaluated by the univariate Cox regression analysis. The multivariable Cox regression analysis included variables that were significantly associated with outcome in the univariate analysis. HR with 95% CI were reported  All statistical tests were two-sided, and *p*-values smaller than 0.05 were considered statistically significant | 3 (IQR 2–4) | 66.1 (IQR  60.41–78.8) |
| Shah 2021 [26]  CIBMTR 2003–2018^†^ | US | 2003–2018 | Median follow-up of survivors was 69 months (range 5–123) and 53 months (range 10–149) in the no early chemo-immunotherapy and the early chemo-immunotherapy groups | 249 | Auto-HCT | CIBMTR (>380 transplant centers worldwide) | Patients with DLBCL who achieved a PR in response to chemotherapy, and subsequently undergoing an auto-HCT | The distributions of OS and PFS were estimated using the Kaplan-Meier method. Cox proportional hazards analysis was used to identify prognostic factors for relapse, NRM, PFS, and OS using forward stepwise variable selection. Covariates with a *p*-value <0.05 were considered statistically significant | **2 (1–5)^§^** | No ECF: 63 (39–77); ECF: 57 (20–76) |
| Buecklein 2020 [27]^‡^ | Germany | January 2019 and June 2020 | Median follow-up: 7 months | 48 | Axi-cel and tisa-cel | Single-center experience of patients with DLBCL treated with axi-cel or tisa-cel at the LMU Munich University Hospital | Patients with DLBCL treated with axi-cel or tisa-cel | NR | 4 (2–9) | 60 (19–74) |
| Dujmovic 2020 [28]^‡^ | Croatia | July 2017–December 2019 | Median follow-up: 6 months | 23 | PoV with immunochemotherapy | Chart review from nine hematology centers in Croatia | Patients with R/R DLBCL treated with PoV | NR | **3 (1–5)^§^** | 64 (39–79) |
| González-Barca 2020 [29]  EBMT 2003–2013^†^ | US | January 2003–December 2013 | Median follow-up of alive patients: 40 months (IQR 22.6–62.6) | 256 | Platinum-containing regimens, active intensive combinations (doxorubicin, cytosine arabinoside, ifosfamide, and gemcitabine), active nonintensive combination | EBMT database/lymphoma registry files and extended by a specific questionnaire | Patients with DLBCL who relapsed after auto-HSCT | Kaplan-Meier method and the log-rank test were used to identify survival differences between subgroups. All analyses were performed at a 95% CI and differences were considered statistically significant when the *p*-value was <0.05 | Number of prior  lines of therapy:  **1: 78 (30%)^§^**  **2: 128 (50%)^§^**  3: 38 (15%)  ≥4: 12 (5%) | 51 (19–70) |
| Tsai 2020 [30]^‡^  National Taiwan University Hospital 2018–2020^†^ | Taiwan | Nov 2018–Apr 2020 | Median follow-up: 11.4 months (range 0.3–20.7) | 32 | PoV combined with other salvage therapy (bendamustine and rituximab, rituximab, rituximab/carmustine-containing regimens, rituximab/gemcitabine regimen) | National Taiwan University Hospital | Patients with DLBCL who developed progressive diseases after at least two prior lines of therapy | NR | **4 (1–7) ^§^** | 62.5 (20–82) |
| Khouri 2020 [31]^‡^  MD Anderson Cancer Center^†^ | US | NR | Median follow-up among survivors: 64.9 months (range 12–190.4) | 59 | AlloSCT | MD Anderson Cancer Center | Patients with R/R transformed indolent B-NHL who underwent alloSCT | Cox regression | **4 (range NR)^§^** | 54 (21.4–66.5) |
| Manji 2020 [32]^‡^ | Canada | 2011–2018 | NR | 90 | NR | NR | Patients with DLBCL who had R/R disease after two lines of therapy | NR | Number of prior  lines of therapy:  2: 90 (100%) | Median age at  second  relapse: 60.4 (IQR  54.7–67.6);  median age at  diagnosis:  58.2 (54.0–  65.0) |
| Mei 2022 [33]  CIBMTR 2003–2017^†^ | US | 2003–2017 | Median follow-up of survivors: 72 months (range 4–145) | 285 | Auto-HCT | CIBMTR registry | Patients with DLBCL who received ≥3 lines of systemic therapy | The Cox proportional hazards model for PFS and OS and the cause-specific hazards model for relapse and NRM were used to identify prognostic factors using forward stepwise variable selection. Results were reported as HR, 95% CI for HR and *p*-value. The adjusted probabilities for each outcome were calculated based on the final regression model. Covariates with a *p*-value <0.05 were considered statistically significant. All statistical analyses were performed using SAS version 9.4 | Number of prior  lines of therapy:  3: 217 (76%)  >3: 68 (24%) | 60 (19–80) |
| Mesguich 2020 [34] | France | January 2008–May 2016 | Median follow-up: 53 months (95% CI: 47–57) | 62 | High-dose chemotherapy followed by ASCT | Department of Hematology of the University Hospital of Bordeaux | Patients with R/R DLBCL treated with R-DHAC salvage therapy and scheduled for HDT-ASCT after at least one course of chemotherapy | Cox proportional hazards models were employed to calculate the HRs of OS/PFS predictors. A two-sided *p*-value <0.05 was considered to reflect statistical significance | **2 (1–3)^§^** | 55 (23–66) |
| Modi 2020 [35] | US | January 2005–December 2017 | Median follow-up of surviving patients of 38 and 67 months for R-BEAM and RIC groups | 70 | R-BEAM/RIC | Karmanos Cancer Institute | Patients with DLBCL who underwent related or unrelated alloSCT for primary induction failure or relapsed DLBCL | Univariable and multivariable Cox proportional hazards regression models were fit to assess associations between four prior chosen predictors (Karnofsky performance status at admission, disease status at transplant, donor type, and group) and survival benefit (RFS and OS) | **3 (1–6)^§^** | 53.5 (25–68) |
| Nydegger 2020 [36]^‡^ | Switzerland | NR | NR | 36 | Axi-cel, tisa-cel, and lisocabtagene maraleucel | NR | Patients with R/R DLBCL | Univariate analysis | Patients with R/R DLBCL  undergoing  CAR-T cell treatment | NR |
| Quivoron 2020 [37]^‡^ | France | 2013–2020 | NR | 89 | NR | NR | Patients with R/R DLBCL | As an exploratory analysis, univariate prognostic analysis for OS was performed | **2 (1–9)^§^** | Mean age 62 years (23–83) |
| Di Rocco 2019 [38]^‡^  One Italian center 2010–2018^†^ | Italy | January 2010 to May 2018 | NR | 116 | NR | One Italian center; retrospective database/clinical data | Patients with R/R DLBCL potentially eligible, according to the approval criteria, for CAR-T cell therapy | Cox proportional hazards model | Number of prior  lines of therapy:  **2: 82 (71%)^§^** | 64 (21–87) |
| Frank 2019 [39]^‡^ | US | NR | Median follow-up: 7.5 months | 54 | Axi-cel | NR | Patients with R/R DLBCL | Response assessed per Lugano criteria | Number of prior  lines of therapy:  **≥3: 59% (range 1–6)^§^** | 61 (19–76) |
| Kittai 2019 [40]^‡^ | US | NR | Median follow-up: 5.06 months | 59 | CAR-T cell therapy: tisa-cel and axi-cel | Four academic medical centers | Patients with R/R DLBCL | Cox proportional hazards models | 3 (2–6) | 63 (25–82) |
| Segman 2019 [41]^‡^  Israeli centers 2018–2019^†^ | Israel | 6/2018–5/2019 | Median follow-up from PoV administration: 4.4 months (range 0.6-11.4) | 34 | PoV | 11 Israeli centers | Patients with R/R DLBCL, treated with Pola-BR or Pola-R (2–8 cycles) | Cox regression model | 3 (3–5) | 65.5 (60–72) |
| Wang 2019 [42]^‡^  MD Anderson Cancer Center^†^ | US | NR | Median follow-up among survivors: 50 months (range 4–217) | 303 | ASCT | MD Anderson Cancer Center | Patients with relapsed DLBCL who underwent ASCT | NR | **2 (range NR)^§^** | 60 (18–80) |
| Ying 2019 [43] | China | November 2010–December 2017 | NR | 84 | Auto-HCT | Peking University Cancer Hospital | Patients with DLBCL | Kaplan-Meier method and compared using the log-rank test. Prognostic factor analysis was performed by the Cox regression model. The limit of statistical significance for all analyses was defined as a *p*-value of <0.05 | Number of prior  lines of therapy:  **1: 42 (50.0%)**  ≥2: 42 (50.0%) | Mean 40.23  (SD 12.57) |
| Myers 2018 [44]  CIBMTR 1990–2008^†^ | US and Canada | January 1, 1990 to December 31, 2008 | Median follow-up: 127 months (range 24–292) | 781 | Auto-HCT | CIBMTR | AYAs and older adults (age >39 years) who had survived progression-free for ≥2 years after auto-HCT for cHL or DLBCL | Cox proportional hazards analysis was used to identify multivariate risk factors. The stepwise selection method with a significance level of 0.05 was used to identify multivariate risk factors | Number of prior lines of therapy:  **1: 134 (17%)^§^**  2: 356 (46%)  3:197 (25%)  >3: 63 (8%) | 51 (15–77) |
| Myers 2017 [45]^‡^ |  |  |  |  |  |  |  |  |  |  |
| Herrera 2017 [46] | US | January 2000–December 2012 | Median follow-up: 45 months (range 7–115) | 117 | ASCT | DFCI/BWH; Boston, MA and COH National Medical Center; Duarte, CA | Patients with chemotherapy-sensitive R/R DLBCL | Cox proportional hazards models with forward variable selection (entry criterion *p*< 0.05) were used to evaluate predictors of PFS and OS. *p*-values were two-sided with a significance level of 0.05 | ASCT: non-DHL/non-DEL: 2  (2–5)  ASCT: DEL (non-DHL): 2–4  ASCT: DHL: 2 (2–3) | ASCT: non-DHL/non-DEL: 58 (30–  73)  ASCT: DEL  (non-DHL): 61 (36–76)  ASCT: DHL:  62 (39–72) |
| Cerrada 2017 [47]^‡^  MD Anderson Cancer Center  2005–2015^†^ | US | 2005–2015 | Median follow-up: 60 months (range 5–120) | 72 | HDT with R-BEAM and ASCT | MD Anderson Cancer Center | Patients with relapsed or primary refractory de novo DLBCL | NR | Number of prior  lines of therapy:  **ABC, >2: 76% GCB, >2: 48%^§^** | 63 (28–74) |
| Van Den Neste 2017 [48]  CORAL; NCT00137995^†^ | Australia, Belgium, Czechia, Finland, Germany, Israel, Sweden, Switzerland, UK, US | July 2003 to June 2008 | Median follow-up: 32.8 months (range 24.3–45.8) | 75 | ICE-type (17.3%), DHAP-type (24%), gemcitabine-containing (28%),  CHOP-like (13.3%), and miscellaneous regimens (17.3%) | The CORAL study | Patients aged 18–65 years with relapsed DLBCL, who relapsed after ASCT | Cox regression analysis was used to calculate the HR between different patient categories. All reported *p*-values are two-sided, and *p*-values <0.05 were considered significant | Number of prior  lines of therapy:  2: 75 (100%) | 56.1 (20.9–  67.7) |
| Van Den Neste 2016 [49]  CORAL; NCT00137995^†^ | Australia, Belgium, Czechia, Finland, Germany, Israel, Sweden, Switzerland, UK, US | July 2003 to June 2008 | Median follow-up: 30.1 months | 203 | Third-line regimens: ICE-type (18.5%), DHAP-type (18%), gemcitabine-containing (13.8%), dexa-BEAM (9%), CHOP-like regimens (8.4%), and miscellaneous regimens (31.9%) | The CORAL study | Patients aged 18–65 years with previously treated DLBCL, who fail second-line salvage regimens in the international CORAL study | Cox regression analysis was used to calculate the HR between different patient categories. All reported *p*-values are two-sided, and *p* < 0.05 was considered significant | Number of prior  lines of therapy:  2: 203 (100%) | 55 (19–65) |
| Eyre 2016 [50] | UK | April 2014–October 2015 | NR | 90 | Pixantrone | Paper and electronic clinical notes | Patients with a R/R DLBCL | Independent predictors were identified using multivariate Cox regression. All univariate predictors with *p* < 0.2 were eligible for inclusion in the initial multivariate model, followed by backward selection with the Akaike information criterion | **2 (1–6)^§^** | 65.9 (20.3–  85.9) |
| Fenske 2016 [51]  CIBMTR 2000–2012^†^ | US | 2000–2012 | Median follow-up of survivors: 55 months (range 11–49) | 503 | Allo-HCT | CIBMTR, TED, CRF | Patients with R/R DLBCL undergoing allo-HCT after experiencing a relapse or progression following a prior auto-HCT | Associations among patient-, disease-, and transplantation-related variables and outcomes of interest were evaluated using Cox proportional hazards regression  Backward elimination was used to identify covariates that influenced outcomes. Covariates with a *p* < 0.05 were considered significant | **4 (1–7)^§^** | 52 (19–72) |
| Gil-Cupello 2016 [52]^‡^ | US | 2005–2011 | Median follow-up in surviving patients: 42.1 months (12.8–80.2) | 34 | ASCT | Institutional transplant registry | Patients who underwent ASCT at a single institution | Univariate Cox proportional hazards regression assessed the relationship between covariates, OS, and PFS. Univariate competing risks regression with Fine and Grey methods assessed the relationship between covariates and time to CR | **2 (0–8)^§^** | 59 (26–72) |
| Kettle 2016 [53]^‡^ | US | 2005–2016 | NR | 43 | ASCT | Winship Cancer Institute, Emory University | Patients with DLBCL receiving >1 salvage therapies | Univariate Cox proportional hazards models of PFS and OS were fit and Kaplan-Meier plots were developed to estimate the impact of variables of interest on survival | All patients received  either two (*n* = 39) or three (*n* = 4) salvage  therapies before  ASCT | 51 |
| Robinson 2016 [54]  EBMT 2002–2009^†^ | Europe | 2002–2009 | Median follow-up in surviving patients: 18 (range 4–48) and 49.5 (range 10–106) | 6947 | ASCT | EBMT database | Patients with R/R DLBCL | The influence of transplant epoch and other potential risk factors on these outcomes was assessed using multivariate Cox proportional hazards regression analysis. All *p*-values were two-sided | ASCT diagnosis  2002–2009:  **1: 5%^§^**  2: 67%  >2: 28%  ASCT diagnosis  1992–2001:  **1: 23%^§^**  2: 57%  >2: 19%  MAC alloSCT:  **1: 4%^§^**  2: 36%  >2: 60%  RIC alloSCT:  2: 23%  >2: 77% | ASCT  diagnosis  2002–2009:  49 (IQR 40–  56);  ASCT  diagnosis  1992–2001:  47 (IQR 38–  54)  MAC alloSCT:  40 (IQR 31–  48);  RIC alloSCT:  50 (IQR 41–  54) |

Abbreviations: ABC = activated B cell; alloSCT = allogeneic stem cell transplant; allo-HCT = allogeneic hematopoietic cell transplant; ASCT = autologous stem cell transplant; auto-HCT = autologous hematopoietic cell transplant; axi-cel = axicabtagene ciloleucel; AYAs = adolescents and young adults; B-NHL = B-cell non-Hodgkin lymphoma; CAR = chimeric antigen receptor; CD19 = cluster of differentiation 19; cHL = classic Hodgkin lymphoma; CHOP = cyclophosphamide, doxorubicin, vincristine, and prednisolone; CI = confidence interval; CIBMTR = Center for International Blood and Marrow Transplant Research; COH = City of Hope; CR = complete response; CRF = comprehensive report form; DEL = double-expressor lymphoma; DFCI/BWH = Dana-Farber Cancer Institute/Brigham and Women’s Hospital; DHL = double-hit lymphoma; DLBCL = diffuse large B-cell lymphoma; EBMT = European Society for Blood and Marrow Transplantation; ECF = early chemo-immunotherapy failure; GCB = germinal center B cell; HDT-ASCT = high-dose chemotherapy followed by autologous stem cell transplant; HR = hazard ratio; ICE = ifosfamide, carboplatinum, etoposide; IQR = interquartile range; ITT = intent-to-treat; LASSO = Least Absolute Shrinkage and Selection Operator; MAC = myeloablative conditioning; NR = not reported; NRM = non-relapse mortality; ORR = overall response rate; OS = overall survival; PFS = progression-free survival; Pola‐BR = polatuzumab vedotin, bendamustine, and rituximab; Pola‐R = polatuzumab vedotin and rituximab; PoV = polatuzumab vedotin; PR = partial response; pRT = palliative radiotherapy; R-BEAM = rituximab, carmustine, etoposide, cytarabine, and melphalan; RCT = randomized controlled trial; R-DHAC = rituximab, dexamethasone, cytarabine, and carboplatin; R±DHAP = rituximab, dexamethasone, cytarabine, and cisplatin; RFS = relapse-free survival; R-GEMOX = rituximab, gemcitabine, oxaliplatin, and dexamethasone; RIC = reduced-intensity conditioning; R/R = relapsed/refractory; SD = standard deviation; TED = transplant essential data; tisa-cel = tisagenlecleucel; UK = United Kingdom; US = United States.

^†^Some studies involved patients from the same trial or data source, and they were presented individually since potentially different subpopulations and analyses were involved. The included population could be overlapping among these studies.

^‡^Conference abstract.

^§^This systematic literature review included patients with DLBCL who failed at least two lines of prior therapy. Bold text indicates that the study had a mixed population involving some patients who received only one line of prior therapy but had ≥50% who received at least two lines of prior therapy or had a median/mean of at least two lines of prior therapy.

**TABLE S2** Patient demographics, clinical characteristics, disease characteristics, treatment characteristics, and imaging and laboratory measures that have a statistically significant association with a clinical outcome.

| **Variable** | **Clinical outcomes** | **Author, year** | **Parameter (vs. reference)**  – category with favorable outcome labeled in green | **N for parameter/ reference** | **Effect estimates**  **(95% CI)** | ***p*-value** | **Supportive evidence** | **Statistical method** | **Multivariate/univariate analysis** | **Study design** | **Publication type** |
| --- | --- | --- | --- | --- | --- | --- | --- | --- | --- | --- | --- |
| ***ECOG PS: higher ECOG PS was associated with worse outcomes in nine studies*** | | | | | | | | | | | |
| ECOG PS | OS | Manji 2020 [32] | ≥2 (vs. <2) | 90 total | HR: 2.68 (1.65–4.34) | <0.05 | “Worse survival was observed in patients with an ECOG greater than or equal to 2” | NR | Not specified | Observational | Conference abstract |
|  |  | Quivoron 2020 [37]^†^ | ≥2 (vs. 0–1) | 89 total | NR | <0.0001 | “A shorter OS was associated with ECOG PS= 2 vs. 0–1” | NR | Univariate | Observational | Conference abstract |
|  |  | Dujmovic 2020 [28]^†^ | 0–2 (vs. 3–4) | 23 total | NR | 0.005 | “Patients with PS 0–2 had significantly better OS (median 12.5 m vs. 4.6 m) than those with PS 3–4” | NR | Not specified | Observational | Conference abstract |
|  |  | Cohen 2022 [17] | >1 (vs. ≤1) | 48 total | HR: 5.5 (1.1–31.0) | 0.04 | “ECOG score >1 is an independent prognostic factor for OS” | Log-rank test and Cox regression | Multivariate | Observational | Journal article |
|  |  | Segman 2021 [25] | ≥2 (vs. <2) | 47 total | HR: 3.86 (1.03–14.46) | 0.045 | “Univariate and multivariate analyses showed the ECOG PS ≥2 was associated with shorter OS” | Cox regression | Univariate | Observational | Journal article |
|  |  | Di Rocco 2021 [20]  Four Italian centers 2010–2018^‡^ | ≥2 (vs. <2) | 137 total | HR: 2.83 (1.66–4.84) | <0.001 | “Multivariate analysis identified ECOG ≥2 as significant independent prognostic factors for OS” | Cox proportional hazards model | Multivariate | Observational | Journal article |
|  |  | Di Rocco 2019 [38]  One Italian center 2010–2018^‡^ | ≥2 (vs. <2) | 116 total | NR | <0.001 | “Multivariate analysis identified ECOG ≥2 as significant prognostic factors for OS” | Cox proportional hazards model | Multivariate | Observational | Journal article |
|  |  | Eastman 2021 [21]^†^ | 2 (vs. 0–1) | 82 total | HR: 1.38 | NR | “The LASSO method selected 5-covariates for the multivariable OS model, which was associated with an optimism-adjusted concordance index of 0.70 (95% CI: 0.61–0.80)” | Cox proportional hazards regression | Multivariate | Observational | Conference abstract |
|  |  |  | 3–4 (vs. 0–1) | 82 total | HR: 5.91 | NR |  |  |  |  |  |
|  |  | Fried 2021 [22] | ≥2 (vs. <2) | 37 total | HR: 6.5 (1.9–22.5) | NR | “In a multivariable ITT Cox regression, factors associated with a shorter OS were ECOG ≥2” | Cox regression | Multivariate | Observational | Conference abstract |
|  | PFS | Dujmovic 2020 [28]^†^ | 0–2 (vs. 3–4) | 23 total | NR | 0.004 | “Patients with PS 0–2 had significantly better PFS (median 9 m vs. 4 m) than those with PS 3–4” | NR | Not specified | Observational | Conference abstract |
| ***Age: older age was associated with worse outcomes in five studies; however, superior OS, PFS, and CR were observed with older age in two studies*** | | | | | | | | | | | |
| Age | OS | Casasnovas 2022 [2]  SADAL^‡^ | <65 years (vs. ≥65 years) | 134 total | NR | 0.037 | “The OS was higher in the <65-year-olds: 13.7 vs. 7.8 months” | Log-rank test and Cox proportional hazards model | Not specified | Randomized controlled trial | Journal article |
|  |  | Zijlstra 2022 [6]  SADAL^‡^ | ≥65 years (vs. <65 years) | 52/82 | HR: 1.7 (1.05–2.78) | 0.03 | “Multivariate analysis including age showed that only age <65 years was independently associated with higher OS” | Log-rank test and Cox proportional hazards model | Multivariate | Randomized controlled trial | Journal article |
|  |  | Cerrada 2017 [47]^†^ | Over 70 years (vs. younger) | 72 total | HR: 4.3 (1.9–9.8) | <0.001 | “Patients over 70 years had significantly worse OS compared to younger patients” | NR | Not specified | Observational | Conference abstract |
|  |  | Hu 2021 [23]^†^ | >60 years (vs. ≤60 years) | 235 total | HR: 1.92 (1.06–3.47) | 0.03 | “In multivariate analysis, age >60 at ASCT remained significant for worse OS” | Cox proportional hazards regression | Multivariate | Observational | Conference abstract |
|  |  | Kittai 2019 [40]^§^ | Older (vs. younger) | 59 total | HR: 0.59 | 0.007 | “Older age was associated with superior OS” | Cox proportional hazards models | Multivariate | Observational | Conference abstract |
|  | PFS | Kittai 2019 [40]^§^ | Older (vs. younger) | 59 total | HR: 0.54 | 0.001 | “Older age was associated with superior PFS” | Cox proportional hazards models | Multivariate | Observational | Conference abstract |
|  | CR | Hu 2021 [7]^¶^ | >50 years (vs. younger) | 20 total | NR | 0.005 | “Subgroups analysis revealed that a higher CR rate was observed in patients over 50 years old compared to younger patients” | Subgroup analysis | Not specified | Randomized controlled trial | Journal article |
|  | NRM | Myers 2018 [44]^†^ | 40–54 years (vs. 15–39 years) | 277/192 | RR: 1.79 (1.08–2.96) | 0.02 | Data not interpreted in text | Cox proportional hazards analysis | Multivariate | Observational | Journal article |
|  |  |  | ≥55 years (vs. 15–39 years) | 312/192 | RR: 4.22 (2.60–6.85) | <0.01 |  |  |  |  |  |
|  | Overall mortality |  | 40–54 years (vs. 15–39 years) | 277/192 | RR: 2.02 (1.32–3.10) | <0.01 |  |  |  |  |  |
|  |  |  | ≥55 years (vs. 15–39 years) | 312/192 | RR: 4.23 (2.80–6.39) | <0.01 |  |  |  |  |  |
| ***KPS: lower KPS was associated with worse outcomes in three studies*** | | | | | | | | | | | |
| KPS | OS | Fenske 2016 [51]^†^ | <80% (vs. 80–100%) | 52/393 | HR: 1.86 (1.33–2.60) | 0.0003 | “On multivariate analysis a higher risk of mortality (inferior OS) was associated with KPS <80” | Cox proportional hazards regression | Multivariate | Observational | Journal article |
|  |  | González-Barca 2020 [29]^†^ | <80% (vs. ≥80%) | 256 total | HR: 1.69 (1.26–2.32) | 0.002 | Data not interpreted in text | Cox regression model | Univariate | Observational | Journal article |
|  |  | Modi 2020 [35]^†^ | Admittance Karnofsky performance status (as continuous variable) | 70 total | HR: 0.960 (0.922–0.999) | 0.044 | “Multivariable analysis revealed that lower Karnofsky performance status was associated with worse OS” | Cox proportional hazards regression | Univariate | Observational | Journal article |
|  | PFS | Fenske 2016 [51]^†^ | <80 (vs. 80–100) | 51/388 | HR: 1.79 (1.29–2.48) | 0.0005 | “Patients with KPS <80 had a higher risk of therapy failure (inferior PFS)” | Cox proportional hazards regression | Multivariate | Observational | Journal article |
|  | Progression/ relapse |  | <80 (vs. 80–100) | 51/388 | HR: 1.81 (1.18–2.77) | 0.006 | “Multivariate analysis demonstrated that KPS <80 was associated with a higher risk of progression/relapse post allo-HCT” |  |  |  |  |
| ***Clinical characteristics reported in one study*** | | | | | | | | | | | |
| Presence of comorbidities by CIRS | OS | Kittai 2019 [40] | Significant comorbidities (vs. without significant comorbidities)  *[Significant comorbidities: CIRS total score ≥7 or CIRS score of 3 or 4 in ≥1 organ system/CIRS-3+]* | 43/16 | HR: 15.44 | 0.042 | “Presence of comorbidities by Cumulative illness rating scale (CIRS) retained independent significance in multivariate models of OS when adjusted with all variables (HR: 15.44, *p* = 0.042)” | Cox proportional hazards models | Multivariate | Observational | Conference abstract |
|  | PFS |  | Significant comorbidities (vs. without significant comorbidities) | 43/16 | HR: 8.64 | 0.002 | “Presence of comorbidities by Cumulative illness rating scale (CIRS) retained independent significance in multivariate models of PFS when adjusted with all variables (HR: 8.64, *p* = 0.002)” | Cox proportional hazards models | Multivariate | Observational | Conference abstract |
| ***Refractory disease: refractory disease compared with non-refractory/relapsed disease was associated with worse outcomes in 10 studies*** | | | | | | | | | | | |
| Refractory disease | OS | Búa 2021 [13]^†^ | Refractory disease (vs. non‐refractory disease)  *[<PR to last regimen]* | 39/25 | HR: 2.2 (1.2–4.2) | 0.016 | “In the multivariate analysis, status of lymphoma (refractory vs. non‐refractory) significantly influenced OS independently from the IPI and the number of previous treatment lines” | Cox model | Multivariate | Non-randomized trial | Conference abstract |
|  |  | Fried 2021 [22] | Primary refractory disease (vs. others^¶^)  *[No definition]* | 37 total | HR: 3.3 (1.2–9.1) | NR | “In a multivariable ITT Cox regression, factors associated with a shorter OS were primary refractory disease” | Cox regression | Multivariate | Observational | Conference abstract |
|  |  | Segman 2021 [25]  Israeli centers 2018–2019^‡^ | Primary refractory disease (vs. relapsed disease)  *[At treatment administration]* | 47 total | HR: 3.766 (1.574–9.008) | 0.003 | Data not interpreted in text | Cox regression | Univariate | Observational | Journal article |
|  |  | Manji 2020 [32] | Refractory disease (vs. relapsed disease)  *[No definition]* | 90 total | HR: 2.61 (1.58–4.32) | <0.05 | “Worse survival was observed in patients with refractory disease” | NR | Not specified | Observational | Conference abstract |
|  |  | Khouri 2020 [31]^†^ | Refractory disease (vs. others^¶^)  *[At time of transplantation]* | 59 total | HR: 5.25 | 0.042 | “By multivariable analysis, disease refractoriness was the only significant factor for a worse OS” | Cox regression | Multivariate | Observational | Conference abstract |
|  | PFS | Búa 2021 [13]^†^ | Refractory disease (vs. non‐refractory disease)  *[<PR to last regimen]* | 39/25 | HR: 3.03 (1.6–5.7) | <0.01 | “Lymphoma status (refractory vs. relapsed) significantly influenced PFS independently from the IPI and the number of previous t/t lines” | Cox model | Multivariate | Non-randomized trial | Conference abstract |
|  |  | Northend 2021 [24]^†^ | Refractoriness to last t/t (vs. others^d^) | 133 total | HR: 3.48 (1.79–6.76) | <0.001 | “Significant factors for shortened PFS by univariate analysis was refractoriness to last t/t” | NR | Univariate | Observational | Conference abstract |
|  |  | Segman 2021 [25]  Israeli centers 2018–2019^‡^ | Primary refractory disease (vs. relapsed disease)  *[At treatment administration]* | 25/22 | HR: 3.049 (1.246–7.463) | 0.015 | “Multivariate analysis confirmed primary refractory disease as the only statistically significant factor associated with a shorter PFS” | Cox regression | Multivariate | Observational | Journal article |
|  |  | Segman 2019 [41]  Israeli centers 2018–2019^‡^ | Primary refractory disease (vs. relapsed disease)  *[No definition]* | 34 total | HR: 1.507 | 0.049 | “Primary refractory vs. relapsed disease tended to be associated with shorter time to progression” | Cox regression model | Univariate | Observational | Conference abstract |
|  |  | Khouri 2020 [31]^†^ | Refractory disease (vs. others^¶^)  *[At time of transplantation]* | 59 total | HR: 3.43 | 0.037 | “By multivariable analysis, disease refractoriness was the only significant factor for a worse PFS” | Cox regression | Multivariate | Observational | Conference abstract |
|  |  | Eyre 2016 [50]^†^ | Relapsed (vs. refractory)  *[Refractory: relapsed within 8 months following a prior PR/CR vs. SD/PD to the immediate prior line of t/t]* | 13/75 | HR: 0.43 (0.22–0.82) | 0.011 | “Patients with relapsed disease had significantly improved PFS” | Cox regression | Multivariate | Observational | Journal article |
|  |  |  | Primary refractory (vs. non-primary refractory)  *[Primary refractory: length of first remission <12 months]* | 90 total | HR: 2.13 (1.31–3.48) | 0.002 | “Patients with non-primary refractory DLBCL (for primary refractory: HR: 2.13 (95% CI: 1.31–3.48, *p*= 0.002) had significantly improved PFS” | Cox regression | Multivariate | Observational | Journal article |
|  |  | Kettle 2016 [53] | Chemotherapy-sensitive disease (vs. refractory disease)  *[No definition]* | 43 total | HR: 0.30 | 0.008 | “Patients who had a chemo-sensitive disease at transplant had a median PFS of 22.6 m; however, patients with refractory disease at transplant only achieved a median PFS of 3.6 m” | Cox proportional hazards | Univariate | Observational | Conference abstract |
|  | CR | Hu 2021 [7] | Relapsed patients (vs. primary refractory patients)  *[No definition]* | 20 total | NR | 0.031 | “Subgroups analysis revealed that relapsed patients had a better CR rate than primary refractory patients” | NR | Univariate | Randomized controlled trial | Journal article |
|  |  | Búa 2021 [13]^†^ | Refractory disease (vs. non‐refractory disease)  *[<PR to last regimen]* | 64 total | NR | <0.01 | “Patients with non-refractory disease had significantly higher CR rate than patients with refractory diseases” | NR | Not specified | Non-randomized trial | Conference abstract |
|  | ORR | Búa 2021 [13]^†^ | Refractory disease (vs. non‐refractory disease)  *[<PR to last regimen]* | 64 total | NR | 0.003 | “Patients with non-refractory disease had significantly higher ORR than patients with refractory diseases” |  |  |  |  |
|  |  | Segman 2021 [25] | Primary refractory (vs. relapsed)  *[At treatment administration]* | 47 total | NR | 0.039 | “Primary refractory compared to relapsed disease was associated with a lower ORR” | Chi-square and Fisher’s exact tests | Univariate | Observational | Journal article |
| ***Response to current therapy: not achieving CR/PR to current therapy was associated with worse OS in seven studies; not achieving CR to current therapy after 6 months was associated with worse PFS in one study*** | | | | | | | | | | | |
| Response to current therapy | OS | Casasnovas 2022 [2]  SADAL^b^ | Never had a CR/PR on prior therapy and achieved CR/PR on selinexor | 134 total | NR | 0.0401 | Data not interpreted in text | Log-rank test and Cox proportional hazards model | Not specified | Randomized controlled trial | Journal article |
|  |  |  | Never had a CR on prior therapy and achieved CR on selinexor | 134 total | NR | 0.0109 |  |  |  |  |  |
|  |  |  | Patients with CR or PR (vs. non-responders)  *[Response to current therapy]* | 39/95 | NR | <0.0001 | “Patients with a best response of CR or PR on selinexor had a markedly longer median OS compared to those who did not respond” |  |  |  |  |
|  |  | Kalakonda 2020 [9]  SADAL^‡^ | ≥PR (vs. ≤SD)  *[Response to current therapy]* | 127 total | NR | <0.0001 | “Median OS was not reached in pts. ≥PR and was 4.9 months in pts ≤SD” | Two-proportion z-test was used to test two group proportions | Not specified | Randomized controlled trial | Journal article |
|  |  | Maerevoet 2018 [11]  SADAL^‡^ | ≥PR (vs. ≤SD)  *[Response to current therapy]* | 11/21 | NR | <0.001 | “Median OS in pts ≥PR was not reached and was significantly longer vs. median OS for pts ≤SD of 4.1 months” | NR | Not specified | Randomized controlled trial | Conference abstract |
|  |  | Segman 2021 [25] | No response (SD/PD) (vs. objective response [CR/PR])  *[Response to current therapy]* | 18/29 | HR: 2.94 (1.09–7.94) | 0.034 | “Response to therapy was associated with a longer OS (14.8 vs. 5.17 months)” | Cox regression | Univariate | Observational | Journal article |
|  |  | Bajwa 2021 [19]^†^ | PD (vs. CR/PR/SD)  *[Response to current therapy on first scan]* | 111 total | HR: 10.1 (4.7–22) | <0.001 | “Accounting for statistically important factors on multivariate analysis, PD on first scan was an independent predictor of OS” | Cox model | Multivariate | Observational | Conference abstract |
|  |  | Tsai 2020 [30]^†^  National Taiwan University Hospital 2018–2020^‡^ | CR or PR (vs. PD)  *[Response to current therapy]* | 14/17 | NR | 0.0001 | “The patients achieving CR or PR after PoV had better OS than those who did not (median OS: not reached vs. 5.6 months)” | NR | Not specified | Observational | Conference abstract |
|  |  | Wang 2022 [18]  National Taiwan University Hospital 2018–2021^‡^ | CR or PR (vs. PD)  *[Response to current therapy]* | 21/18 | HR: 0.115 (0.044–0.301) | <0.001 | “The median OS was significantly improved in patients who achieved CR or PR compared with those who did not (24.0 months vs. 4.2 months, *p* < 0.001)” | Log-rank test | Univariate | Observational | Journal article |
|  |  |  | CR or PR (vs. others)  *[Response to current therapy in patients receiving subsequent HSCT]* | 2/12 | HR: 0.165 (0.027–1.003) | 0.026 | “For patients who received subsequent HSCT, those who achieved CR/PR after PoV-based treatment had a significantly better OS” |  |  |  |  |
|  | PFS | Nydegger 2020 [36] | CR after 6 months (vs. others)  *[Response to current therapy]* | 11/14 | NR | <0.0001 | “Parameters associated with better outcome were achievement of CR after 6 months in the univariate analysis” | NR | Univariate | Observational | Conference abstract |
| ***Response to therapy prior to SCT: not achieving CR to therapy prior to SCT was associated with worse outcomes in five studies*** | | | | | | | | | | | |
| Response to therapy prior to SCT; remission status at SCT | OS | Hu 2021 [23]^†^ | Non-CR (vs. CR)  *[At transplantation]* | 235 total | HR: 2.22 (1.26–3.90) | <0.01 | “In multivariate analysis, non-CR at auto-HCT remained significant for worse OS” | Cox proportional hazards regression | Multivariate | Observational | Conference abstract |
|  |  | Mei 2022 [33]  CIBMTR 2003–2017^‡^ | PR (vs. CR)  *[At transplantation]* | 131/154 | HR: 1.55 (1.12–2.15) | 0.009 | “PR at auto-HCT was associated with significantly inferior OS” | Cox proportional hazards model | Multivariate | Observational | Journal article |
|  |  | Fenske 2016 [51]^†^  CIBMTR 2000–2012^‡^ | Chemotherapy resistant (vs. CR)  *[Remission status at HCT]* | 106/175 | HR: 1.94 (1.44–2.62) | <0.0001 | “On multivariate analysis a higher risk of mortality (inferior OS) was associated with chemo-resistant disease” | Cox proportional hazards regression | Multivariate | Observational | Journal article |
|  |  | Herrera 2017 [46] | PR (vs. CR)  *[At transplantation]* | 117 total | HR: 2.4 (1.3–4.4) | 0.007 | “Factor significantly associated with OS was remission status at ASCT” | Cox proportional hazards models | Multivariate | Observational | Journal article |
|  |  | Gil-Cupello 2016 [52]^†^ | CR (vs. others^¶^)  *[Pre-autologous SCT]* | 34 total | HR: 0.24 (0.1–0.56) | 0.001 | “CR pre-ASCT was significantly associated with OS” | Cox proportional hazards regression | Univariate | Observational | Conference abstract |
|  | PFS | Mei 2022 [33]  CIBMTR 2003–2017^‡^ | PR (vs. CR)  *[At transplantation]* | 131/154 | HR: 1.46 (1.08–1.97) | 0.01 | “PR at auto-HCT was associated with significantly inferior PFS” | Cox proportional hazards model | Multivariate | Observational | Journal article |
|  |  | Fenske 2016 [51]^†^  CIBMTR 2000–2012^‡^ | Chemotherapy resistant (vs. CR)  *[Remission status at HCT]* | 104/173 | HR: 2.04 (1.53–2.73) | <0.0001 | “Patients with chemo-resistant disease had a higher risk of therapy failure (inferior PFS)” | Cox proportional hazards regression | Multivariate | Observational | Journal article |
|  |  | Gil-Cupello 2016 [52]^†^ | CR (vs. others^¶^)  *[Pre-ASCT]* | 34 total | HR: 0.44 (0.21–0.92) | 0.03 | “CR pre-autologous SCT was significantly associated with PFS” | Cox proportional hazards regression | Univariate | Observational | Conference abstract |
|  | Progression/ relapse | Mei 2022 [33]  CIBMTR 2003–2017^‡^ | PR (vs. CR)  *[At transplantation]* | 131/154 | HR: 1.59 (1.13–2.24) | 0.008 | “PR at auto-HCT was associated with significantly increased risk of relapse” | Cox proportional hazards model | Multivariate | Observational | Journal article |
|  |  | Fenske 2016 [51]^†^  CIBMTR 2000–2012^‡^ | Chemotherapy resistant (vs. CR)  *[Remission status at HCT]* | 104/173 | HR: 2.25 (1.51–3.36) | <0.0001 | “Multivariate analysis for demonstrated that chemo-resistant disease was associated with a higher risk of progression/relapse post allo-HCT” | Cox proportional hazards regression | Multivariate | Observational | Journal article |
|  | NRM | Fenske 2016 [51]^†^ | Chemotherapy resistant (vs. CR)  *[Remission status at HCT]* | 104/173 | HR: 1.86 (1.23–2.81) | 0.003 | “On multivariate analysis, chemo-resistant disease before HCT was associated with a higher risk of NRM” | Cox proportional hazards regression | Multivariate | Observational | Journal article |
|  | CR (after relapse/ progression) | Gil-Cupello 2016 [52]^†^ | CR (vs. others^¶^)  *[Pre-ASCT]* | 34 total | HR: 6.06 (1.42–25.88) | 0.015 | “CR after REL/POD… and CR pre-ASCT (HR: 6.06, 95% CI: 1.42–25.88, *p*= 0.015), …, were significantly associated” | Competing risks regression with Fine and Grey methods assessed the relationship between covariates and time to CR | Univariate | Observational | Conference abstract |
| ***Response to prior therapy: not achieving response to prior third-line regimen was associated with worse OS in one study*** | | | | | | | | | | | |
| Response to prior therapy | OS | Van Den Neste 2017 [48] | CR/CRu (vs. SD/PD)  *[To third-line regimen]* | 75 total | HR: 0.104 | <0.0001 | “Achievement of CR/CRu independently predicted for OS” | Cox regression analysis | Multivariate | Observational | Journal article |
|  |  |  | PR (vs. SD/PD)  *[To third-line regimen]* | 75 total | HR: 0.242 | 0.0186 | “Achievement of PR independently predicted for OS” |  |  |  |  |
| ***R-IPI/t-IPI: higher IPI score was associated with worse outcomes in five studies*** | | | | | | | | | | | |
| IPI | OS | Van Den Neste 2017 [48]  CORAL^‡^ | >2 (vs. 0–2)  *[At relapse post-ASCT (t-IPI)]* | 75 total | HR: 2.464 | 0.0139 | “t-IPI >2, independently predicted for OS” | Cox regression analysis | Multivariate | Observational | Journal article |
|  |  | Van Den Neste 2016 [49]  CORAL^‡^ | >2 (vs. 0–2)  *[At relapse and transplantation]* | 203 total | HR: 2.74 | <0.0001 | “t-IPI >2 was independently associated with OS” | Cox regression analysis | Multivariate | Observational | Journal article |
|  |  | Wang 2022 [18] | High risk (vs. low and intermediate risk)  *[At diagnosis]* | 28/9 | HR: 4.582 (1.349–15.560) | 0.008 | “R-IPI risk stratification could be used to effectively predict OS not only at diagnosis but also at the time of PoV t/t (low/intermediate-risk vs. high-risk groups: median at diagnosis, not reached vs. 6.2 m, *p* = 0.008)” | Log-rank test | Univariate | Observational | Journal article |
|  |  |  | High risk (vs. low and intermediate risk)  *[At the time of PoV treatment]* | 28/12 | HR: 3.773 (1.404–10.140) | 0.005 | “R-IPI risk stratification could be used to effectively predict OS not only at diagnosis but also at the time of PoV t/t (low/intermediate-risk vs. high-risk groups: median at the time of PoV t/t, 24 m vs. 5.6 m)” |  |  |  |  |
|  |  | Casasnovas 2022 [2] | 0–2 (vs. 3–5)  *[No definition]* | 134 total | HR: 0.38 (0.24–0.60) | <0.0001 | “Patients with lower baseline R-IPI (0–2) compared to R-IPI (3–5) had a significantly longer median OS (15.1 vs. 4.6 m)” | Log-rank test and Cox proportional hazards model | Not specified | Randomized controlled trial | Journal article |
|  |  | Bajwa 2021 [19]^†^ | 4–5 (vs. 0–1)  *[No definition]* | 111 total | HR: 4.05 (1.26–13.02) | 0.02 | Data not interpreted in text | Cox model | Multivariate | Observational | Conference abstract |
|  | ORR | Wang 2022 [18] | ≥3 (vs. <3)  *[Before PoV-based salvage treatment and before PoV administration]* | 40 total | NR | 0.014 | “Patients who had R-IPI scores ≥3 before PoV-based salvage t/t had a lower ORR” | Kruskal–Wallis and chi-square tests | Not specified | Observational | Journal article |
| ***Prior lines of therapy: greater number of prior lines of therapy was associated with worse outcomes in four studies*** | | | | | | | | | | | |
| Prior lines of therapy | OS | Eastman 2021 [21]^†^ | Per one-unit increase  *[Continuous]* | 82 total | HR: 1.23 | NR | “The LASSO method selected 5-covariates for the multivariable OS model, which was associated with an optimism-adjusted concordance index of 0.70 (95% CI: 0.61–0.80)” | Cox proportional hazards regression | Multivariate | Observational | Conference abstract |
|  | PFS | Northend 2021 [24]^†^ | ≥2 (vs. <2) | 133 total | HR: 2.17 (1.19–3.95) | 0.01 | “Significant factors for shortened PFS by univariate analysis was ≥2 prior treatments” | NR | Univariate | Observational | Conference abstract |
|  |  | Eyre 2016 [50]^†^ | Per extra line of therapy/treatment  *[Continuous]* | 90 total | HR: 1.66 (1.29–2.14) | <0.001 | “Those with fewer lines of prior therapy had significantly improved PFS” | Cox regression | Multivariate | Observational | Journal article |
|  |  | Ying 2019 [43] | ≥2 (vs. 1) | 84 total | HR: 18.065 (2.38–137.105) | 0.005 | “The number of previous regimens administered prior to HSCT was associated with PFS” | Log-rank test and Cox regression model | Not specified | Observational | Journal article |
| ***Double-expressor or double-hit lymphoma: double-expressor or double-hit lymphoma was associated with worse outcomes in four studies*** | | | | | | | | | | | |
| Double- expressor or double-hit lymphoma | OS | Casasnovas 2022 [2]  SADAL^‡^ | Non-DE DLBCL (vs. DE DLBCL)  *[DE DLBCL was defined as C-MYC overexpression (>40% of tumor cells stained positive for C-MYC) and BCL2 overexpression (>50% of tumor cells stained positive for BCL2)]* | 134 total | HR: 0.43 (0.23–0.77) | 0.004 | “DLBCL which was not DE was associated with a significantly longer median OS (13.7 months [95% CI: 11.1–32.3] vs. 5.1 months [95% CI: 3.0–15.1]” | Log-rank test and Cox proportional hazards model | Not specified | Randomized controlled trial | Journal article |
|  |  | Herrera 2017 [46] | DHL (vs. neither DEL nor DHL)  *[DHL was defined as concurrent rearrangements of MYC and BCL2 and/or BCL6; DEL was defined as MYC expression in ≥40% of tumor cells and BCL2 expression in ≥50% of tumor cells]* | 12/58 | HR: 3.4 (1.5–7.6) | 0.004 | “Factor significantly associated with OS was DHL” | Cox proportional hazards models | Multivariate | Observational | Journal article |
|  |  | Wang 2019 [42]^†^ | DEL/DHL (vs. non-DEL/non-DHL; DEL)  *[DEL was defined as dual expression of MYC and BCL2; cutoff values of 40% for MYC and 70% for BCL2 were established by IHC. DHL was defined as MYC, BCL2, and/or BCL6 translocations]* | 303 total | NR | 0.034 | “There was no significant difference in OS between non-DEL/non-DHL and DEL subgroups (*p* = 0.39); however, a significant difference in OS was observed between the two subgroups compared to the DEL/DHL patients” | NR | Not specified | Observational | Conference abstract |
|  | PFS | Herrera 2017 [46] | DEL (vs. neither DEL nor DHL)  *[DEL was defined as MYC expression in ≥40% of tumor cells and BCL2 expression in ≥50% of tumor cells]* | 47/58 | HR: 1.8 (1.05–3.2) | 0.035 | “Factor significantly associated with PFS was DEL” | Cox proportional hazards models | Multivariate | Observational | Journal article |
|  |  |  | DHL (vs. neither DEL nor DHL)  *[DHL was defined as concurrent rearrangements of MYC and BCL2 and/or BCL6]* | 12/58 | HR: 2.9 (1.3–6.3) | 0.009 | “Factor significantly associated with PFS was DHL” |  |  |  |  |
|  | ORR | Casasnovas 2022 [2]  SADAL^‡^ | Non-DE DLBCL (vs. DE DLBCL)  *[DE DLBCL was defined as C-MYC overexpression (>40% of tumor cells stained positive for C-MYC) and BCL2 overexpression (>50% of tumor cells stained positive for BCL2)]* | 134 total | NR | 0.012 | “DLBCL which was not DE was associated with a significantly higher ORR than DE disease (46.2% vs. 14.8%)” | Two-proportion z-test was used to test two group proportions | Not specified | Randomized controlled trial | Journal article |
|  |  | Kalakonda 2020 [9]  SADAL^‡^ | Double or triple expressor (vs. no)  *[Double- or triple- expressor status were determined by IHC; further definition not specified]* | 31/57 | NR | 0.0056 | “Similar results were observed with ORR for DLBCL with double or triple expressor status (3/31; 9.7%, 95% CI: 2.0–25.8), for DLBCL without double or triple expressor status (23/57; 40.3%, 27.6–54.2; *p* = 0.0056) but these differences were largely reflection of c-MYC overexpression because expression levels of neither Bcl-2 nor Bcl-6 affected the ORR” | Two-proportion z-test was used to test two group proportions | Not specified | Randomized controlled trial | Journal article |
|  | NRM | Wang 2019 [42]^†^ | Both DEL and DHL (vs. non-DEL/non-DHL; DEL)  *[DEL was defined as dual expression of MYC and BCL2; cutoff values of 40% for MYC and 70% for BCL2 were established by IHC. DHL was defined as MYC, BCL2, and/or BCL6 translocations]* | 303 total | HR: 3.8 | 0.017 | “A higher risk of NRM was observed in the DEL/DHL group compared to the other two groups” | NR | Not specified | Observational | Conference abstract |
| ***MYC overexpression or mutation: overexpression or mutation of MYC/c-MYC was associated with worse OS and ORR in three studies*** | | | | | | | | | | | |
| MYC overexpression or mutation | OS | Quivoron 2020 [37]^†^ | MYC mutation (vs. others)  *[MYC mutation; further definition not specified]* | 89 total | NR | 0.0220 | “Among the most recurrently altered genes, a shorter OS was associated with MYC mutation (*p* = 0.0220)” | Exploratory analysis | Univariate | Observational | Conference abstract |
|  |  | Kittai 2019 [40] | MYC rearrangement (positive on fluorescence in situ hybridization vs. negative) | 59 total | HR: 3.23 | 0.032 | “MYC also retained independent significance regarding OS” | Cox proportional hazards models | Multivariate | Observational | Conference abstract |
|  | ORR | Kalakonda 2020 [9] | High levels of c-MYC (≥40% positive cells)  (vs. c-MYC <40% positive cells) | 47/52 | NR | 0.0024 | “Patients with high levels of c-MYC (based on a cutoff of 40% positive cells as determined by IHC) had a 13% ORR (6/47; 95% CI: 4.8–25.7), whereas those with low levels had a 42% (22/52; 28.7–56.8) ORR (*p* = 0.0024)” | Two-proportion z-test was used to test two group proportions | Not specified | Randomized controlled trial | Journal article |

| ***Transformed disease: transformed disease was associated with worse OS and DOR in two studies, but better PFS in one study*** | | | | | | | | | | | |
| --- | --- | --- | --- | --- | --- | --- | --- | --- | --- | --- | --- |
| Transformed disease^§^ | OS | Segman 2019 [41] | Transformed (vs. de novo)  *[t-FL]* | 34 total | NR | 0.008 | “T-FL vs. de novo DLBCL was associated with decreased OS” | Cox regression model | Univariate | Observational | Conference abstract |
|  | PFS | Nydegger 2020 [36] | Transformed (vs. de novo)  *[No definition]* | 19/20 | NR | 0.034 | “Parameters associated with better outcome were transformed versus de novo disease in the univariate analysis” | NR | Univariate | Observational | Conference abstract |
|  | DOR | Casasnovas 2022 [2] | de novo (vs. transformed)  *[Transformed from previously diagnosed indolent lymphoma but all included patients with transformed DLBCL had a history of follicular lymphoma]* | 134 total | HR: 0.2 (0.06–0.64) | 0.003 | “The median DOR was significantly longer in patients with de novo DLBCL (23 months [95% CI: 9.7–not reached] vs. 4.4 months [95% CI: 2.0–9.2])” | Log-rank test and Cox proportional hazards model | Not specified | Randomized controlled trial | Journal article |
| ***Cell of origin: non-GCB DLBCL was associated with worse OS in one study; however, higher ORR was associated with non-GCB DLBCL in another study*** | | | | | | | | | | | |
| COO^§^ | OS | Hu 2021 [23]^†^ | Non-GCB (vs. GCB) | 235 total | HR: 1.81 (1.03–3.18) | 0.04 | “In multivariate analysis, non-GCB COO remained significant for worse OS” | Cox proportional hazards regression | Multivariate | Observational | Conference abstract |
|  | ORR | Wang 2022 [18] | Non-GCB (vs. GCB) | 40 total | NR | 0.044 | “Patients with non-GCB DLBCL had a higher ORR than those with GCB DLBCL (65.0% vs. 31.3%)” | Kruskal–Wallis and chi-square tests | Not specified | Observational | Journal article |
| ***Ann Arbor stage: higher Ann Arbor stage was associated with worse outcomes in two studies*** | | | | | | | | | | | |
| Ann Arbor stage | OS | Quivoron 2020 [37]^†^ | III–IV (vs. I–II) | 89 total | NR | 0.0023 | “A shorter OS was associated with Ann Arbor stage III–IV vs. I–II” | NR | Univariate | Observational | Conference abstract |
|  |  | Di Rocco 2021 [20] | III–IV (vs. I–II) | 137 total | HR: 2.01 (1.13–3.57) | 0.017 | “Multivariate analysis identified Ann Arbor stage III–IV as significant independent prognostic factors for OS” | Cox proportional hazards model | Multivariate | Observational | Journal article |
| ***Disease bulk: presence of bulky disease and disease bulk of >7.5 cm was associated with worse outcomes in two studies*** | | | | | | | | | | | |
| Disease bulk | PFS | Northend 2021 [24]^†^ | >7.5cm (vs. ≤7.5cm) | 133 total | HR: 2.32 (1.23–4.38) | 0.009 | “Significant factors for shortened PFS by univariate analysis was bulk disease >7.5cm” | NR | Univariate | Observational | Conference abstract |
|  | ORR | Graf 2018 [16] | Yes (vs. no) | 17 total | NR | 0.04 | “ORR was associated with disease bulk, with 60% ORR in non-bulky vs. 0% ORR in cases of bulky disease” | NR | Not specified | Non-randomized trial | Conference abstract |
| ***Conditional regimen: TBI and myeloablative conditioning was associated with worse outcomes in two studies*** | | | | | | | | | | | |
| TBI conditioning | NRM | Myers 2018 [44]^†^  CIBMTR 1990–2008^‡^ | Yes (vs. no) | 115/666 | RR: 1.51 (1.01–2.25) | 0.043 | Data not interpreted in text | Cox proportional hazards regression | Multivariate | Observational | Journal article |
|  | Overall mortality |  | Yes (vs. no) | 115/666 | RR: 1.51 (1.09–2.08) | 0.013 |  |  |  |  |  |
| Conditioning regimen | OS | Fenske 2016 [51]^†^  CIBMTR 2000–2012^‡^ | Myeloablative (vs. reduced-intensity conditioning/non-myeloablative conditioning) | 127/376 | HR: 1.39 (1.09–1.78) | 0.008 | “On multivariate analysis a higher risk of mortality (inferior OS) was associated with myeloablative conditioning” | Cox proportional hazards regression | Multivariate | Observational | Journal article |
|  | PFS |  | Myeloablative (vs. reduced-intensity conditioning/non-myeloablative conditioning) | 126/368 | HR: 1.29 (1.09–1.63) | 0.03 | “Patients with use of myeloablative conditioning had a higher risk of therapy failure (inferior PFS)” |  |  |  |  |
|  | NRM |  | Myeloablative (vs. reduced-intensity conditioning/non-myeloablative conditioning) | 126/368 | HR: 1.99 (1.34–2.95) | 0.001 | “During the first 10 months post-transplant it was associated with a higher NRM, but not beyond 10 months post-allo-HCT” |  |  |  |  |
| ***Prior ASCT: prior ability to receive ASCT was associated with better outcomes in two studies*** | | | | | | | | | | | |
| Prior ASCT | OS | Van Den Neste 2016 [49] | No (vs. yes) | 203 total | HR: 2.667 | 0.0002 | “Ability to perform transplantation was independently associated with OS (*p* = 0.0002, HR: 2.667)” | Cox regression analysis | Multivariate | Observational | Journal article |
|  | PFS | Graf 2021 [12] | Yes (vs. no) | 8/10 | NR | <0.01 | “Prior receipt of ASCT, possibly reflecting selection of patients who are more fit and/or have less t/t-resistant disease, was also associated with improved median PFS (6.2 vs. 2.3 m; *p* < 0.01)” | NR | Univariate | Non-randomized trial | Journal article |
|  | ORR |  | Yes (vs. no) | 8/10 | NR | <0.01 | “Prior receipt of ASCT, possibly reflecting selection of patients who are more fit and/or have less t/t-resistant disease, was also associated with improved ORR (75% vs. 10%; *p* = 0.01)” |  |  |  |  |
| ***Time to REL/POD: shorter time from ASCT to REL/POD was associated with worse OS, PFS, and CR in two studies*** | | | | | | | | | | | |
| Time from ASCT to REL/POD | OS | González-Barca 2020 [29]^†^ | >1 year (vs. ≤1 year)  *[Time from first ASCT to relapse]* | 90/166 | HR: 0.52 (0.38–0.71) | <0.0001 | Data not interpreted in text | Cox regression model | Univariate | Observational | Journal article |
|  |  | Gil-Cupello 2016 [52] | Days from ASCT to REL/POD  *[Continuous]* | 34 total | HR: 0.95 (0.91–0.99) | 0.01 | “Days from ASCT to REL/POD was significantly associated with OS” | Cox proportional hazards regression | Univariate | Observational | Conference abstract |
|  | PFS | Gil-Cupello 2016 [52] | Days from ASCT to REL/POD  *[Continuous]* | 34 total | HR: 0.95 (0.91–0.99) | 0.008 | “Days from ASCT to REL/POD was significantly associated with PFS” | Cox proportional hazards regression | Univariate | Observational | Conference abstract |
|  | CR (after REL/POD) |  | Days from ASCT to REL/POD  *[Continuous]* | 34 total | HR: 1.04 (1.01–1.07) | 0.008 | “CR after REL/POD… and days from ASCT to REL/POD (HR: 1.04, 95% CI: 1.01–1.07, *p* = 0.012), …, were significantly associated” | Competing risks regression with Fine and Grey methods assessed the relationship between covariates and time to CR | Univariate | Observational | Conference abstract |
| ***Time from diagnosis: shorter time from diagnosis to treatment was associated with lower ORR and CR in two studies*** | | | | | | | | | | | |
| Time from diagnosis | ORR | Segman 2021 [25] | Shorter (vs. longer) | 47 total | NR | 0.019 | “Shorter time from diagnosis was associated with a lower ORR” | Chi-square and Fisher’s exact tests | Univariate | Observational | Journal article |
|  | CR (after REL/POD) | Gil-Cupello 2016 [52] | Days from diagnosis to ASCT  *[Continuous]* | 34 total | HR: 1.04 (1.02–1.06) | <0.0001 | “CR after REL/POD… and days from diagnosis to ASCT (HR: 1.04, 95% CI: 1.02–1.06, *p* < 0.0001), …, were significantly associated” | Competing risks regression with Fine and Grey methods assessed the relationship between covariates and time to CR | Univariate | Observational | Conference abstract |
| ***Disease and treatment characteristics reported in one study*** | | | | | | | | | | | |
| Tumor burden | PFS | Graf 2021 [12] | Low (vs. high)  *[High: MDT ≥4 cm or MTV ≥100 cm^3^]* | 9/9 | NR | <0.01 | “Low burden of disease by MDT or MTV was associated with… longer median PFS (6.2 vs. 2.4 months; *p* < 0.01)” | Exploratory analyses | Univariate | Non-randomized trial | Journal article |
|  | ORR |  | Low (vs. high) | 9/9 | NR | 0.05 | “Univariate analyses showed low burden of disease by MDT or MTV was associated with a higher ORR (66% vs. 11%; *p* = 0.05)” |  |  |  |  |
| BCL2 expression | OS | Wang 2019 [42]^†^ | Positive (vs. negative) | 303 total | HR: 1.82 | 0.049 | “Patients who had BCL2 (+) expression, had worse OS (HR: 1.82; *p* = 0.049)” | NR | Not specified | Observational | Conference abstract |
|  |  |  | BCL2 negative (vs. BCL2 positive)  *[Among GCB subtype]* | 18/111 | NR | 0.03 | “The 4-year OS rates of GCB/BCL2(-) and GCB/BCL2(+) were 87% and 56%, respectively (*p*= 0.030)” |  |  |  |  |
|  | PFS |  | BCL2 negative (vs. BCL2 positive)  *[Among GCB subtype]* | 18/111 | NR | 0.007 | “The 4-year PFS rates were 88% and 47%, respectively (*p* = 0.007)” |  |  |  |  |
| Year of diagnosis of DLBCL | OS | Robinson 2016 [54]^†^ | After 2002 (vs. before 2002) | 6947 total | HR: 0.85 (0.78–0.92) | <0.0001 | “After multivariate analysis adjusting for confounding factors, a diagnosis after 2002 was associated with higher OS after autoSCT” | Cox proportional hazards regression | Multivariate | Observational | Journal article |
|  | PFS |  | After 2002 (vs. before 2002) | 6947 total | HR: 0.91 (0.85–0.97) | 0.005 | “After multivariate analysis adjusting for confounding factors, a diagnosis after 2002 was associated with PFS after autoSCT” |  |  |  |  |
|  | NRM |  | After 2002 (vs. before 2002) | 6947 total | HR: 0.79 (0.66–0.96) | 0.016 | “After multivariate analysis adjusting for confounding factors, a diagnosis after 2002 was associated with a lower NRM” |  |  |  |  |
| B symptoms | OS | Eastman 2021 [21]^†^ | Yes (vs. no) | 82 total | HR: 1.13 | NR | “The LASSO method selected 5-covariates for the multivariable OS model, which was associated with an optimism-adjusted concordance index of 0.70 (95% CI: 0.61–0.80)” | Cox proportional hazards regression | Multivariate | Observational | Conference abstract |
| ECF | OS | Shah 2021 [26]^†^ | Yes (vs. no)  *[ECF: patients with primary refractory disease or relapse within 12 months of diagnosis]* | 182/67 | HR: 1.61 (1.05–2.46) | 0.03 | “On multivariate analysis, ECF patients had an increased risk for death (HR: 1.61; 95% CI: 1.05–2.46; *p* = 0.03)” | Cox proportional hazards analysis | Multivariate | Observational | Journal article |
| Primary intent of radiation therapy | OS | Eastman 2021 [21]^†^ | Palliative symptoms (vs. salvage) | 82 total | HR: 1.92 | NR | “The LASSO method selected 5-covariates for the multivariable OS model, which was associated with an optimism-adjusted concordance index of 0.70 (95% CI: 0.61–0.80)” | Cox proportional hazards regression | Multivariate | Observational | Conference abstract |
|  |  |  | Bridge (vs. salvage) | 82 total | HR: 1.33 | NR |  |  |  |  |  |
| CAR-T therapy eligibility based on clinical trial criteria | OS | Manji 2020 [32] | Eligible (vs. not eligible) | 90 total | HR: 0.41 (0.35–0.68) | <0.05 | “Fig 1; Patients who would have been eligible for CAR-T therapy based on clinical trial criteria had better survival (HR: 0.41, CI: 0.35–0.68, *p* < 0.05)” | NR | Not specified | Observational | Conference abstract |
| Post-ASCT DFI | OS | Van Den Neste 2017 [48] | <6 months (vs. ≥12 months) | 75 total | HR: 2.270 | 0.0497 | “Post-ASCT disease-free interval (DFI) lasting <6 months (HR: 2.270, *p* = 0.0497) independently predicted for OS” | Cox regression analysis | Multivariate | Observational | Journal article |
|  |  |  | <6 months,  6-12 months  (vs. ≥12 months) | 75 total | NR | Global *p*-value 0.0132 | Data not interpreted in text |  |  |  |  |
| Graft type | OS  (during ≤3 months post-transplant) | Fenske 2016 [51]^†^ | Peripheral blood (vs. bone marrow) | 456/47 | HR: 0.37 (0.22–0.61) | <0.0001 | “During the first 3 months post-transplant, peripheral blood grafts were associated with a lower risk of mortality” | Cox proportional hazards regression | Multivariate | Observational | Journal article |
| TIBAA | PFS |  | <12 months (vs. ≥12 months) | 200/294 | HR: 1.32 (1.06–1.64) | 0.01 | “Patients with short TIBAA had a higher risk of therapy failure (inferior PFS)” |  |  |  |  |
|  | Progression/ relapse |  | <12 months (vs. ≥12 months) | 200/294 | HR: 2.28 (1.66–3.14) | <0.0001 | “During the first year post-allo HCT, a short (<12 months) TIBAA was associated with a higher progression/relapse risk” |  |  |  |  |
| Type of donor | NRM |  | URD/HLA-identical sibling (vs. well matched/partially matched) | 249/245 | HR: 1.44 (1.04–2.00) | 0.03 | “On multivariate analysis, URD transplantation was associated with a higher risk of NRM“ |  |  |  |  |
| ***LDH: elevated LDH was associated with worse OS and PFS in six studies; however, it was associated with better OS in one study*** | | | | | | | | | | | |
| LDH^§^ | OS | Zijlstra 2022 [6] | >ULN (vs. ≤ULN) | 62/69 | HR: 2.35 (1.45–3.79) | <0.001 | “Multivariate analysis of LDH showed that only LDH ULN ≤ was independently associated with higher OS” | Cox proportional hazards model | Multivariate | Randomized controlled trial | Journal article |
|  |  | Wang 2022 [18] | >271 IU/mL (vs. ≤271 IU/mL) | 21/19 | HR: 2.859 (1.254–6.516) | 0.009 | “A higher LDH level (>271 IU/mL) before PoV treatment was a poor prognostic factor for OS” | Log-rank test | Univariate | Observational | Journal article |
|  |  | Cohen 2022 [17] | >450 U/I (vs. ≤450 U/I) | 48 total | HR: 7.7 (1.9–32.0) | <0.01 | “Serum LDH >450 U/l is an independent prognostic factor for OS” | Cox regression | Multivariate | Observational | Journal article |
|  |  | González-Barca 2020 [29]^†^ | Elevated (vs. normal) | 256 total | HR: 2.08 (1.49–2.86) | <0.0001 | Data not interpreted in text | Cox regression model | Univariate | Observational | Journal article |
|  |  | Di Rocco 2019 [38]^†^ | Elevated (vs. normal) | 116 total | NR | 0.019 | “Multivariate analysis identified elevated LDH as significant prognostic factors for OS” | Cox proportional hazards model | Multivariate | Observational | Conference abstract |
|  |  | Nydegger 2020 [36] | LDH (as continuous variable) | 26 total | NR | 0.0577 | “Parameters associated with better outcome was LDH (OS; *p* = 0.0577) in the univariate analysis” | Univariate analysis | Univariate | Observational | Conference abstract |
|  | PFS | Buecklein 2020 [27] | Normal (vs. elevated) | 31 total | NR | 0.031 | “PFS was significantly longer for pts with normal vs. elevated LDH at time of apheresis (not reached vs. 1.5 m)” | NR | Not specified | Observational | Conference abstract |
| ***Deauville score: higher Deauville score was associated with worse outcomes in four studies*** | | | | | | | | | | | |
| Deauville score (the Deauville 5-point scale is based on a visual comparison between the uptake of lymphoma tissue and that of the liver and mediastinum in PET/CT) | OS | Cohen 2022 [17] | >3 (vs. ≤3) | 48 total | HR: 7.2 (1.5–34.6) | 0.01 | “Deauville score >3 on M1-PET was the only factor significantly associated with OS” | Log-rank test and univariate Cox regression | Multivariate | Observational | Journal article |
|  |  | Frank 2019 [39]^†^ | PET positive (vs. negative)  *[PET positive: Deauville >3]* | 25/28 | NR | 0.0096 | “When compared to PET-positive patients, PET-negative patients demonstrated an improved median OS (both not reached)” | NR | Not specified | Observational | Conference abstract |
|  |  | Ying 2019 [43]^†^ | Post-HSCT PET positive (vs. negative)  *[PET positive: Deauville >3]* | 84 total | HR: 26.138 (5.607–121.855) | <0.001 | “Post-HSCT PET finding was significantly associated with 3-year OS” | Cox regression model | Multivariate | Observational | Journal article |
|  | PFS | Mesguich 2020 [34]^†^ | Grade 5 (vs. Grade 1–4) | 62 total | HR: 4.92 (1.38–17.49) | 0.01 | “Patients with a Deauville score of 5 on pre-transplant PET/CT had a poorer outcome with a shorter PFS on univariate analysis” | Cox proportional hazards models | Univariate | Observational | Journal article |
|  |  | Frank 2019 [39]^†^ | PET positive (vs. negative)  *[PET positive: Deauville >3]* | 25/28 | NR | 0.0007 | “When compared to PET +ve patients, PET -ve patients demonstrated an improved median PFS (not reached vs. 3.1 months)” | NR | Not specified | Observational | Conference abstract |
|  |  | Ying 2019 [43]^†^ | Post-HSCT PET positive (vs. negative)  *[PET positive: Deauville >3]* | 84 total | HR: 5.168 (2.055–12.999) | <0.001 | “Post-HSCT PET finding was significantly associated with 3-year PFS” | Cox regression model | Multivariate | Observational | Journal article |
| ***SUV^max^: higher value of SUV was associated with worse outcomes in two studies*** | | | | | | | | | | | |
| SUV^max^ on pre-transplant PET/CT | OS | Cohen 2022 [17] | >17.1 (vs. ≤17.1) | 48 total | HR: 10.3 (2.2–47.7) | <0.01 | “TD-SUV^max^ >17.1 is an independent prognostic factor for OS” | Cox regression | Multivariate | Observational | Journal article |
|  | Response | Sang 2020 [15]^†^ | SUV^max^  *[Continuous]* | 21 total | NR | 0.038 | “The SUV^max^ (g/ml) of 15 evaluable patients with treatment response (median of 12.23, range: 6.49–35.71) was significantly lower than that of three patients without response (median of 24.8, range: 18.6–42.29) (*p* = 0.038)” | T test, Wilcoxon signed rank test or Mann-Whitney U test | Not specified | Non-randomized trial | Journal article |
| ***ALC: lower ALC was associated with worse outcome in one study***  ***ALC/AMC ratio: lower ALC/AMC was associated with worse outcome in one study***  ***ALC/AMC prognostic score: high-risk disease according to ALC/AMC prognostic score was associated with worse outcome in one study*** | | | | | | | | | | | |
| ***ALC/AMC*** | OS | Casasnovas 2022 [2] | ALC ≥1000/μL (vs. ALC <1000/μL) | 134 total | NR | 0.013 | “Median OS were 15.5 and 7.6 months in patients with ALC ≥1000/μL and <1000/μL, *p* = 0.013” | Log-rank test and Cox proportional hazards model | Not specified | Randomized controlled trial | Journal article |
|  |  | Wang 2022 [18] | ALC/AMC ratio >1.5 (vs. ≤1.5) | 20/20 | HR: 0.3702 (0.1619–0.8465) | 0.014 | “Patients with an ALC/AMC ratio >1.5 had significantly longer OS than those with a ratio ≤1.5” | Log-rank test | Univariate | Observational | Journal article |
|  | ORR |  | ALC/AMC prognostic score as high risk (vs. low risk; intermediate risk) | 40 total | NR | 0.027 | “ALC/AMC prognostic score predict the ORR effectively in different risk groups (high-risk, 22.2%; intermediate-risk, 57.8%; and low-risk, 100%)” | Kruskal–Wallis and chi-square tests | Not specified | Observational | Journal article |
| ***Laboratory measures reported in one study*** | | | | | | | | | | | |
| TLG value on pre-transplant PET/CT | OS | Mesguich 2020 [34]^†^ | TLG value  *[Continuous]* | 62 total | HR: 1.01 (1.001–1.013) | 0.03 | “The pre-transplant TLG value (median = 20; range 0–432) was the only parameter associated with an increased risk of OS in our study, and was, in fact, the most powerful prognostic factor” | Cox proportional hazards models | Univariate | Observational | Journal article |
|  | PFS |  | High (vs. low) | 62 total | HR: 6.61 (1.55–28.2) | 0.01 | “After adjustment for the International Prognostic Index (IPI) score, multivariate analysis showed that a high TLG value remained significantly associated with PFS” |  | Multivariate |  |  |
| MRD detected by circulating tumor DNA | OS | Frank 2019 [39] | Positive (vs. negative)  *[Positive: any detectable circulating tumor DNA]* | 28/22 | NR | 0.0005 | “As compared to MRD-pos, MRD neg correlated with improved median OS (not reached vs.7.4 months, *p* = 0.0005)” | NR | Not specified | Observational | Conference abstract |
|  | PFS |  | Positive (vs. negative)  *[Positive: any detectable circulating tumor DNA]* | 28/22 | NR | <0.0001 | “As compared to MRD-pos, MRD neg correlated with improved median PFS (not reached vs. 2.96 months, *p* < 0.0001)” | NR | Not specified | Observational | Conference abstract |

Abbreviations: ALC/AMC = absolute lymphocyte count/absolute monocyte count; allo-HCT = allogenic hematopoietic cell transplant; ASCT = autologous stem cell transplant; auto-HCT = autologous hematopoietic cell transplant; BCL2 = B-cell lymphoma 2; CAR = chimeric antigen receptor; CI = confidence interval; CIBMTR = Center for International Blood and Marrow Transplant Research; CIRS = Cumulative illness rating scale; COO = cell of origin; CR = complete response; CRu = complete response unconfirmed; CT = computed tomography; DE = double expressor; DEL = double-expressor lymphoma; DFI, disease-free interval; DHL = double-hit lymphoma; DLBCL = diffuse large B-cell lymphoma; DOR = duration of response; ECF = early chemo-immunotherapy failure; ECOG = Eastern Cooperative Oncology Group; GCB = germinal center B cell; HCT = hematopoietic cell transplant; HLA = human leukocyte antigen; HR = hazard ratio; HSCT = hematopoietic stem cell transplant; IHC = immunohistochemistry; IPI = International Prognostic Index; ITT = intent-to-treat; KPS = Karnofsky performance score; LASSO = Least Absolute Shrinkage and Selection Operator; LDH = lactate dehydrogenase; max = maximum; MDT = maximum dimension of largest tumor; MRD = minimal residual disease; MTV = metabolic tumor volume; NR = not reported; NRM = non-relapse mortality; ORR = overall response rate; OS = overall survival; PD = progressive disease; PET = positron emission tomography; PFS = progression-free survival; POD = progression of disease; PoV = polatuzumab vedotin; PR = partial response; PS = performance status; REL = relapse; R-IPI = revised International Prognostic Index; R/R = relapsed/refractory; RR = risk ratio; SCT = stem cell transplant; SD = stable disease; SUV = standardized uptake value; TBI = total body irradiation; T-FL = transformed follicular lymphoma; TIBAA = time interval between auto-HCT and allo-HCT; t-IPI = tertiary International Prognostic Index; TLG = total lesion glycolysis; t/t = treatment; ULN = upper limit of normal; URD = unrelated donor.

^†^This systematic literature review included patients with DLBCL who failed at least two lines of prior therapy. This symbol indicates that the study had a mixed population involving some patients who received only one line of prior therapy but had ≥50% who received at least two lines of prior therapy or had a median/mean of at least two lines of prior therapy.

^‡^Studies involving the same data sources and may have overlapping population.

^§^Prognostic factor with conflicting directionality across studies.

^¶^Others: comparator was not specified.

**Appendix F – Risk of bias**

**Risk of bias for prognostic factor studies**


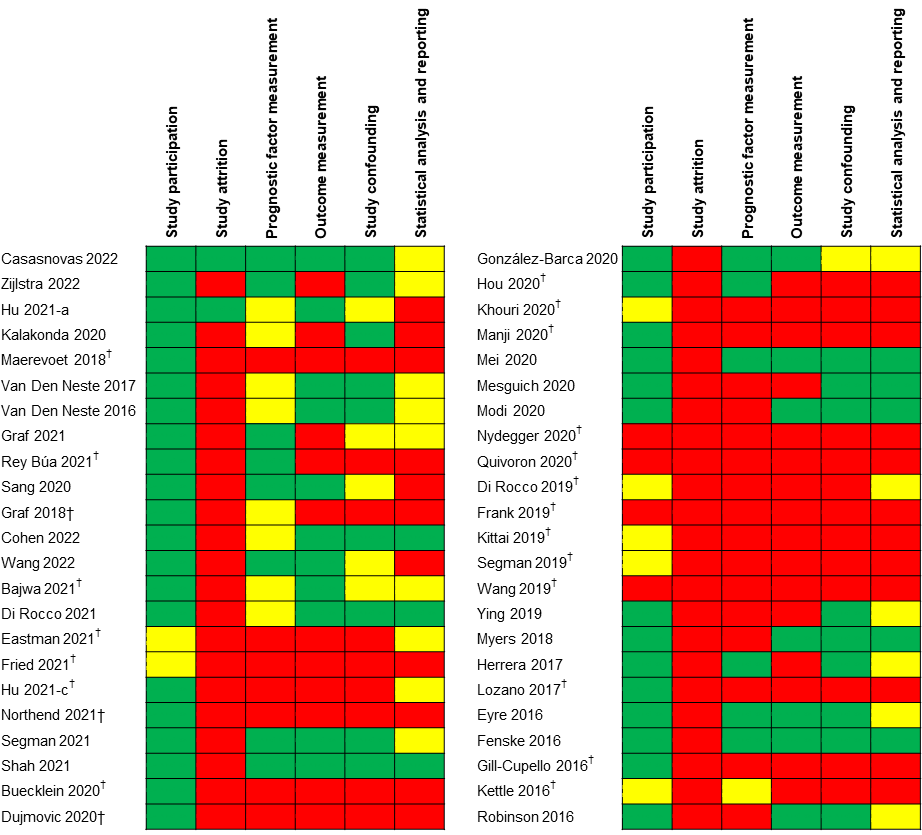


Red = high risk of bias; yellow = moderate risk of bias; green = low risk of bias.

^†^Conference abstract.

**References for supplemental appendix**

1. Hayden, J.A., et al., *Assessing bias in studies of prognostic factors.* Ann Intern Med, 2013. **158**(4): p. 280–6.

2. Casasnovas, R.O., et al., *Comparison of the effectiveness and safety of the oral selective inhibitor of nuclear export, selinexor, in diffuse large B cell lymphoma subtypes.* Clin Lymphoma Myeloma Leuk, 2022. **22**(1): p. 24–33.

3. Maerevoet, M., et al., *Survival among patients with relapsed/refractory diffuse large B cell lymphoma treated with single-agent selinexor in the SADAL study.* J Hematol Oncol, 2021. **14**(1): p. 111.

4. Schuster, M., et al. *Lymphocyte count effect on efficacy and safety of single agent oral selinexor in patients with relapsed/refractory diffuse large B-cell lymphoma (DLBCL): a post-hoc analysis from phase 2B Sadal study*. in *European Hematology Association (EHA) 2021 Virtual Congress Abstract Book HemaSphere*. 2021.

5. Schuster, M.W., et al., *Effect of age on the efficacy and safety of single agent oral selinexor in patients with relapsed/refractory diffuse large B-cell lymphoma (DLBCL): a post-hoc analysis of the Sadal pivotal study.* Blood, 2020. **136**(Suppl. 1): p. 5–6.

6. Zijlstra, J.M., et al., *The association between patient characteristics and the efficacy and safety of selinexor in diffuse large B-cell lymphoma in the SADAL study.* Cancers, 2022. **14**(3): p. 791.

7. Hu, J., et al., *Combination of decitabine and a modified regimen of cisplatin, cytarabine and dexamethasone: a potential salvage regimen for relapsed or refractory diffuse large B-cell lymphoma after second-line treatment failure.* Front Oncol, 2021. **11**: p. 687374.

8. Hu, J., et al., *Combination of decitabine and modified DHAO regimen: a potential salvage regimen for relapsed/refractory diffuse large B‐cell lymphoma after second‐line treatment failure.* Hematol Oncol, 2021. **39**(Suppl. 2): p. Abstract 325.

9. Kalakonda, N., et al., *Selinexor in patients with relapsed or refractory diffuse large B-cell lymphoma (SADAL): a single-arm, multinational, multicentre, open-label, phase 2 trial.* Lancet Haematol, 2020. **7**(7): p. e511–e522.

10. Zijlstra, J.M., et al. *Efficacy and safety of single agent oral selinexor in patients with primary refractory diffuse large B-cell lymphoma (DLBCL): a post-hoc analysis of the SADAL study*. in *European Hematology Association (EHA) 2020 Virtual Congress Abstract Book HemaSphere*. 2020.

11. Maerevoet, M., et al., *Single agent oral selinexor demonstrates deep and durable responses in relapsed/refractory diffuse large B-cell lymphoma (DLBCL) in both GCB and non-GCB subtypes: the phase 2b Sadal study.* Blood, 2018. **132**(Suppl. 1): p. 1677.

12. Graf, S.A., et al., *Ibrutinib monotherapy in relapsed or refractory, transformed diffuse large B-cell lymphoma.* Clin Lymphoma Myeloma Leuk, 2021. **21**(3): p. 176–181.

13. Búa, B.R., et al., *ABCL-181: Updated results of a phase 2 study from GELTAMO investigating the combination of ibrutinib with R-GEMOX in patients with relapsed or refractory diffuse large B-cell lymphoma.* Clin Lymphoma Myeloma Leuk, 2021. **21**: p. S381.

14. Búa, B.R., et al., *Ibrutinib in combination with R-Gemox-D in patients with relapsed or refractory diffuse large B-cell lymphoma: phase II clinical trial of the Geltamo group.* Blood, 2020. **136**(Suppl. 1): p. 16–17.

15. Sang, W., et al., *Phase II trial of co‐administration of CD19‐and CD20‐targeted chimeric antigen receptor T cells for relapsed and refractory diffuse large B cell lymphoma.* Cancer Med, 2020. **9**(16): p. 5827–5838.

16. Graf, S.A., et al., *Ibrutinib is effective in relapsed or refractory transformed indolent B-cell non-Hodgkin lymphoma: results from a prospective phase II study.* Blood, 2018. **132**(Suppl. 1): p. 2954.

17. Cohen, D., et al., *[^18^F] FDG PET-CT in patients with DLBCL treated with CAR-T cell therapy: a practical approach of reporting pre- and post-treatment studies.* Eur J Nucl Med Mol Imaging, 2022. **49**(3): p. 953–962.

18. Wang, Y.-W., et al., *Polatuzumab vedotin–based salvage immunochemotherapy as third-line or beyond treatment for patients with diffuse large B-cell lymphoma: a real-world experience.* Ann Hematol, 2022. **101**(2): p. 349–358.

19. Bajwa, A., et al., *Prognostic value of early imaging following CAR T-cell therapy in DLBCL.* J Clin Oncol, 2021. **39**(15 Suppl.): p. e19559.

20. Di Rocco, A., et al., *Relapsed/refractory diffuse large B-cell lymphoma patients. A multicenter retrospective analysis of eligibility criteria for car-T cell therapy.* Leuk Lymphoma, 2021. **62**(4): p. 828–836.

21. Eastman, B., et al., *Pilot prognostic model for survival in r/r DLBCL patients receiving palliative radiation therapy.* Int J Radiat Oncol Biol Phys, 2021. **111**(3): p. e299–e300.

22. Fried, S., et al., *Patients with out of specification tisagenlecleucel can be salvaged with a point‐of‐care CAR T‐cells: an observational intention‐to‐treat single‐center analysis.* Hematol Oncol, 2021. **39**(Suppl. 2): p. Abstract 270.

23. Hu, M., et al., *Predictors of relapse and survival following autologous stem cell transplant in patients with diffuse large B-cell lymphoma.* Blood, 2021. **138**(Suppl. 1): p. 1832.

24. Northend, M., et al., *Polatuzumab vedotin with bendamustine and rituximab for relapsed/refractory high‐grade B‐cell lymphoma: the UK experience.* Hematol Oncol, 2021. **39**(Suppl. 2): p. Abstract 174.

25. Segman, Y., et al., *Outcome of relapsed/refractory diffuse large B-cell lymphoma patients treated with polatuzumab vedotin-based therapy: real-life experience.* Leuk Lymphoma, 2021. **62**(1): p. 118–124.

26. Shah, N.N., et al., *Is autologous transplant in relapsed DLBCL patients achieving only a PET+ PR appropriate in the CAR T-cell era?* Blood, 2021. **137**(10): p. 1416–1423.

27. Buecklein, V., et al., *Single-center experience with axicabtagene-ciloleucel (axi-cel) and tisagenlecleucel (tisa-cel) for relapsed/refractory diffuse large B-cell lymphoma: comparable response rates and manageable toxicity.* Blood, 2020. **136**(Suppl. 1): p. 34–35.

28. Dujmovic, D., et al. *Polatuzumab-vedotin combined with immunochemotherapy in R/R patients with DLBCL: a retrospective, non-interventional, real-life study of krohem, the croatian cooperative group for hematologic diseases*. in *European Hematology Association (EHA) 2021 Virtual Congress Abstract Book HemaSphere*. 2020.

29. González-Barca, E., et al., *Outcome in patients with diffuse large B-cell lymphoma who relapse after autologous stem cell transplantation and receive active therapy. A retrospective analysis of the Lymphoma Working Party of the European Society for Blood and Marrow Transplantation (EBMT).* Bone Marrow Transplant, 2020. **55**(2): p. 393–399.

30. Tsai, C.-H., et al., *Polatuzumab vedotin-based salvage chemotherapy in the third-line or above treatment for diffuse large B-cell lymphoma.* Blood, 2020. **136**(Suppl. 1): p. 12.

31. Khouri, I., et al. *Clinical relevance of MYC/BCLl2, cell of origin and conditioning regimen in patients with relapsed diffuse large b-cell lymphoma (DLBCL) treated with allogeneic stem cell transplantation (alloSCT)*. in *European Hematology Association (EHA) 2020 Virtual Congress Abstract Book HemaSphere*. 2020.

32. Manji, F. and D.A. Stewart, *Real world characteristics and outcomes of patients with relapsed and refractory diffuse large B cell lymphoma; a provincial experience.* Blood, 2020. **136**(Suppl. 1): p. 17–18.

33. Mei, M., et al., *Autologous hematopoietic cell transplantation in diffuse large B-cell lymphoma after three or more lines of prior therapy: evidence of durable benefit.* Haematologica, 2022. **107**(5): p. 1214–1217.

34. Mesguich, C., et al., *Prognostic utility of pre‐transplantation [^18^F] fluorodeoxyglucose positron emission tomography/computed tomography in patients with diffuse large B‐cell lymphoma who underwent rituximab, dexamethasone, high‐dose cytarabine, carboplatin salvage chemotherapy.* Br J Haematol, 2020. **188**(2): p. 268–271.

35. Modi, D., et al., *R-BEAM versus reduced-intensity conditioning regimens in patients undergoing allogeneic stem cell transplantation for relapsed refractory diffuse large B cell lymphoma.* Biol Blood Marrow Transplant, 2020. **26**(4): p. 683–690.

36. Nydegger, A., et al. *Analysis of clinical and laboratory parameters associated with outcome after CAR-T treatment in DLBCL patients*. in *Swiss Hematology & Oncology Congress (SOHC)*. 2020.

37. Quivoron, C., et al., *High incidence of TP53 and epigenetic modifying oncogenes mutations in a large cohort of patients enrolled in phase 1 clinical trials for relapsed or refractory diffuse large B-cell lymphoma.* Blood, 2020. **136**(Suppl. 1): p. 10–11.

38. Di Rocco, A., et al., *Relapsed/refractory diffuse large B-cell lymphoma (R/R DLBCL) patients: a retrospective analysis of eligibility criteria for CAR-T cell therapy.* Blood, 2019. **134**(Suppl. 1): p. 2888.

39. Frank, M.J., et al., *Detectable circulating tumor DNA 28 days after the CD19 CAR T-cell therapy, axicabtagene ciloleucel, is associated with poor outcomes in patients with diffuse large B-cell lymphoma.* Blood, 2019. **134**(Suppl. 1): p. 884.

40. Kittai, A.S., et al., *Comorbidities predict inferior survival in patients receiving CAR T-cell therapy for relapsed/refractory DLBCL: a multicenter retrospective analysis.* Blood, 2019. **134**(Suppl. 1): p. 780.

41. Segman, Y., et al., *Outcome of relapsed DLBCL patients, treated with polatuzumab-BR or polatuzumab-R: real life data.* Blood, 2019. **134**(Suppl. 1): p. 5321.

42. Wang, Y., et al., *Clinical relevance of MYC/BCL2 and cell of origin in patients with relapsed diffuse large B-cell lymphoma treated with autologous stem cell transplantation.* Blood, 2019. **134**(Suppl. 1): p. 2021.

43. Ying, Z., et al., *Prognostic value of ^18^F-fluorodeoxyglucose positron emission tomography using Deauville criteria in diffuse large B cell lymphoma treated with autologous hematopoietic stem cell transplantation.* Chin J Cancer Res, 2019. **31**(1): p. 162–170.

44. Myers, R.M., et al., *Long‐term outcomes among 2‐year survivors of autologous hematopoietic cell transplantation for Hodgkin and diffuse large b‐cell lymphoma.* Cancer, 2018. **124**(4): p. 816–825.

45. Myers, R., et al., *Long-term outcomes among two-year survivors of autologous hematopoietic cell transplant for Hodgkin and diffuse large B-cell lymphoma.* Biol Blood Marrow Transplant, 2017. **23**(3): p. S27–S28.

46. Herrera, A.F., et al., *Relapsed or refractory double-expressor and double-hit lymphomas have inferior progression-free survival after autologous stem-cell transplantation.* J Clin Oncol, 2017. **35**(1): p. 24–31.

47. Cerrada, S.L., et al., *The cell of origin has no prognostic impact on high-dose chemotherapy with R-beam and autologous stem cell transplant for diffuse large B cell lymphoma.* Bone Marrow Transplant, 2017. **52**(Suppl. 1): p. 364–365.

48. Van Den Neste, E., et al., *Outcomes of diffuse large B-cell lymphoma patients relapsing after autologous stem cell transplantation: an analysis of patients included in the CORAL study.* Bone Marrow Transplant, 2017. **52**(2): p. 216–221.

49. Van Den Neste, E., et al., *Outcome of patients with relapsed diffuse large B-cell lymphoma who fail second-line salvage regimens in the International CORAL study.* Bone Marrow Transplant, 2016. **51**(1): p. 51–57.

50. Eyre, T.A., et al., *Results of a multicentre UK‐wide retrospective study evaluating the efficacy of pixantrone in relapsed, refractory diffuse large B cell lymphoma.* Br J Haematol, 2016. **173**(6): p. 896–904.

51. Fenske, T.S., et al., *Allogeneic transplantation provides durable remission in a subset of DLBCL patients relapsing after autologous transplantation.* Br J Haematol, 2016. **174**(2): p. 235–248.

52. Gil-Cupello, M., et al., *Outcomes of diffuse large B-cell lymphoma (DLBCL) patients progressing after autologous hematopoietic stem cell transplant.* Biol Blood Marrow Transplant, 2016. **22**(3): p. S232–S233.

53. Kettle, A.G., et al., *Prolonged progression-free survival is possible in patients with diffuse large B-cell lymphoma receiving > 1 salvage therapies before autologous stem cell transplant.* Blood, 2016. **128**(22): p. 5825.

54. Robinson, S.P., et al., *Autologous stem cell transplantation for relapsed/refractory diffuse large B-cell lymphoma: efficacy in the rituximab era and comparison to first allogeneic transplants. A report from the EBMT Lymphoma Working Party.* Bone Marrow Transplant, 2016. **51**(3): p. 365–371.
